# Fundamentals and comprehensive insights on pulsed laser synthesis of advanced materials for diverse photo- and electrocatalytic applications

**DOI:** 10.1038/s41377-022-00904-7

**Published:** 2022-08-10

**Authors:** Jayaraman Theerthagiri, K. Karuppasamy, Seung Jun Lee, R. Shwetharani, Hyun-Seok Kim, S. K. Khadheer Pasha, Muthupandian Ashokkumar, Myong Yong Choi

**Affiliations:** 1 Core-Facility Center for Photochemistry & Nanomaterials, Department of Chemistry, Research Institute of Natural Sciences, Gyeongsang National University, Jinju, 52828 Republic of Korea; 2 Division of Electronics and Electrical Engineering, Dongguk University-Seoul, Seoul, 04620 Republic of Korea; 3 Centre for Nano and Material Sciences, Jain University, Jain Global Campus, Kanakapura, Bangalore, 562112 Karnataka India; 4 Department of Physics, Vellore Institute of Technology (Amaravati Campus), Amaravati, 522501 Guntur, Andhra Pradesh India; 5 School of Chemistry, University of Melbourne, Parkville Campus, Melbourne, VIC 3010 Australia

**Keywords:** Nanoparticles, Laser-produced plasmas

## Abstract

The global energy crisis is increasing the demand for innovative materials with high purity and functionality for the development of clean energy production and storage. The development of novel photo- and electrocatalysts significantly depends on synthetic techniques that facilitate the production of tailored advanced nanomaterials. The emerging use of pulsed laser in liquid synthesis has attracted immense interest as an effective synthetic technology with several advantages over conventional chemical and physical synthetic routes, including the fine-tuning of size, composition, surface, and crystalline structures, and defect densities and is associated with the catalytic, electronic, thermal, optical, and mechanical properties of the produced nanomaterials. Herein, we present an overview of the fundamental understanding and importance of the pulsed laser process, namely various roles and mechanisms involved in the production of various types of nanomaterials, such as metal nanoparticles, oxides, non-oxides, and carbon-based materials. We mainly cover the advancement of photo- and electrocatalytic nanomaterials via pulsed laser-assisted technologies with detailed mechanistic insights and structural optimization along with effective catalytic performances in various energy and environmental remediation processes. Finally, the future directions and challenges of pulsed laser techniques are briefly underlined. This review can exert practical guidance for the future design and fabrication of innovative pulsed laser-induced nanomaterials with fascinating properties for advanced catalysis applications.

## Abstract (web summary)

Pulsed laser method uses a laser as an energy source to produce diverse materials in various environments for the reactions of photo- and electrocatalytic applications.

## Graphical abstract

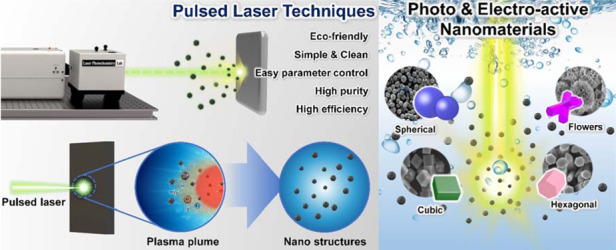

## Introduction

The rapid growth of population and the development of civilization has led to increased energy consumption along with environmental pollution and energy crisis, which has become a global challenge. Subsequently, the exploration of renewable energy has been significantly accelerated to achieve sustainable development. The development of efficient energy conversion and storage devices and environmental remediation technologies based on electro- and photochemistry is of paramount importance to tackle these challenges^1–6^. However, the abovementioned devices function with different working mechanisms, and their reliability and photo- or electrochemical performances are mainly controlled by the involved active catalytic materials. Thus, the design and synthesis of advanced materials and their implications have been exhaustively investigated.

The earlier decade has identified the prompt expansion of nanoscience and nanotechnology, which emerged as a key discipline due to various applications in practically all fields associated with energy and the environment. Nanomaterials are well defined as material configurations with a size scale in the range of 1–100 nm. The concept of nanotechnology was first introduced by Nobel laureate and American physicist Richard Feynman during the annual meeting of the American Physical Society in 1959. The Japanese scientist Norio Taniguchi first coined the term “nanotechnology” in 1974 to depict the specific production method of materials^7,8^. The area of nanotechnology was only briefly discussed until the 1980s, and the concept of nanotechnology has taken ground afterward with the prospective for technological advancements.

Nanomaterials possess unique properties with specific structures, size, surface-area-to-volume ratio, purity, and composition of the materials. Particularly, photo- and electrocatalytic properties can be advanced by controlling the compositions and structures of the nanomaterials. These unique properties make them efficient and flexible to be used for global needs in diverse energy and environmental applications^9–13^. Thus, the uniqueness of nanomaterials originates from their synthesis because even a small modification in the synthetic process can lead to a tremendous change in their intrinsic properties. Numerous methods have focused on the fine-tuned synthesis of nanomaterials using new techniques to accomplish distinct architectures, surface structures, size, stability, and chemical composition. However, nanomaterial production development faces several difficulties. Each synthetic method has its merits and demerits. The traditional synthetic methods are often energy- and time-consuming. Conventional chemical (thermal decomposition, chemical reduction, hydrolysis, solvothermal, hydrothermal, and electrochemical methods) and physical (microwave, sonochemical, chemical vapor deposition (CVD), and physical vapor deposition) methods require expensive precursor materials, involve ligand exchange reactions, and generate toxic byproducts or use stabilizing agents/surfactants^1,8,14,15^. Consequently, the advancement of rapid and facile synthetic techniques for producing highly efficient nanomaterials is expected to overcome the abovementioned issues in conventional synthetic methods and improve the functionality and purity of the products.

In recent decades, pulsed laser-assisted synthesis, which integrates the use of light with nanomaterial synthesis, has drawn numerous attention as an efficient method for the production of several compositions of nano- and submicron-sized materials and holds huge promise for fine-tuning the surface, electronic, and/or crystal structure of nanomaterials. The pulsed laser method uses a laser as an energy source for the reaction of targeted source materials. The interaction of the laser and source materials can be designed in various environments to produce diverse materials^2,16,17^. The pulsed laser-assisted synthetic route offers many degrees of parameter control (i.e., pulsed laser wavelength, power, reaction time duration, laser pulse repetition rate, and solvent) and possesses several advantages over conventional chemical and physical synthetic routes to control the fine-tuning of size, composition, surface, and crystalline structures associated with catalytic, electronic, thermal, optical, and mechanical properties^1,18,19^.

In this review article, we mainly focus on recent advances in photo- and electrocatalytic nanomaterials via pulsed laser-assisted synthesis technologies with detailed mechanistic insights and chemical, physical, and structural properties along with effective catalytic performances in various energy and environmental remediation processes. After a concise research overview, we present the importance of pulsed laser-assisted synthesis methods and fundamental concepts in the pulsed laser process, i.e., various roles and mechanisms involved in the production of various categories of nanomaterials, such as metal nanoparticles (MNPs), oxides, non-oxides, chalcogenides, and carbon-based materials. Consequently, the structural optimization/regulation and versatile applications of laser-assisted catalytic nanomaterials for energy conversion, storage, and environmental remediation applications are comprehensively summarized. Finally, the future directions and challenges in pulsed laser techniques are briefly proposed.

### Advantages of laser-assisted methodologies

1. The pulsed laser-assisted synthesis is a “green chemistry” process. No surfactants or capping agents are required during the synthesis of nanomaterials, whereas most wet chemical synthetic routes involve surfactants and are often incomplete or poorly reproducible.
2. The absence of surfactants and capping agents is advantageous as these molecules can ligate the surface of atoms and block the catalytically surface-active sites of the particles.
3. No byproducts and toxic reducing agents are needed for the reaction. Hence, the pulsed laser synthesis techniques are environmentally friendly.
4. The experimental setup is simple and minimal, offering a low-cost method.
5. Pulsed laser-assisted synthesis of nanomaterials (<100 nm) with uniform size distribution is exceptionally rapid. The production of the nanomaterials in bulk quantities can take approximately 1 h or less, depending on the laser repetition rate. Scale-up production to grams per hour has been demonstrated, making it appropriate for industrial applications^20–22^.
6. The pulsed laser process can also be used for fabricating inorganic metal complexes and metal-organic frameworks (MOF) or the surface modification of nanomaterials, such as nanoparticles coated with organic molecules, which can be rapidly obtained using a single-step process.

**Fig. 1:**
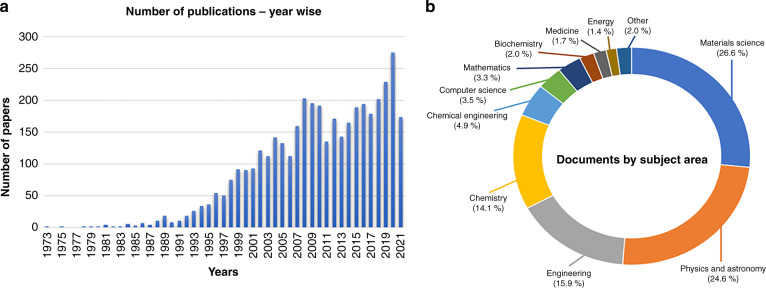
Progress in pulsed laser synthesis. **a** Number of publications in the Scopus database by the search term “pulsed laser synthesis” from 1973 to 2021. **b** Contribution of the works by subject (Source: Scopus database, searched on August 31, 2021)

Owing to the above advantages, research on the production of nanomaterials using pulsed laser-assisted technologies has been increasing. Conducting a literature search on the topic based on the Scopus database using the search term “pulsed laser synthesis,” we found 4074 publications with an increasing publication frequency from 1973 to 2021 (Source: Scopus database, searched on August 31, 2021), as shown in Fig. 1a. Notably, the nanomaterials synthesized by pulsed laser methods are mostly concentrated in the “materials science” subject (Fig. 1b), proposing diverse photo- and electrocatalytic applications.

## Fundamentals of the pulsed laser process

In 1960, Theodore Harold Maiman designed the first functional laser at Hughes Research Laboratories by focusing a high-power flash lamp on the surface of a silver-coated ruby rod^23^. Since then, lasers have been broadly utilized in scientific and technological applications. Through scientific advancements, the interaction of the laser and targeted source materials can be constructed in different environments to produce various functional materials. At the beginning of 1965, the ruby laser was applied in vacuum chambers to produce dielectric thin films, which is called pulsed laser deposition (PLD). In the PLD process, the desired material is vaporized from the source of the target in ultrahigh vacuum conditions to create a plasma plume and then deposited on a substrate as a film. The PLD technique is used to fabricate a variety of thin films, including metallic multilayers, ceramic oxides, nitrides, and various superlattices with fine crystalline quality and different textures. Also, PLD validates relatively less costs compared to other thin film deposition techniques such as CVD and molecular beam epitaxy^2,16^.

Especially, in the last two decades, pulsed laser ablation (PLA) and pulsed laser irradiation (PLI) have been widely applied for the production of various nanostructures. PLA and PLI differ in terms of the interaction of the incident laser beam on the surface of the target source. PLA applies a focus lens at various distances to assemble the laser beam in one spot with ~1 mm size. It can deliver high energy on the designated small spot and leads to target substance evaporation into the solvent to form desired nanostructures. Conversely, the PLI process utilizes no focus lenses and is applied to colloidal solutions rather than solid targets^11^. In general, the formation of NPs in the PLA in the liquid (PLAL) process occurs via two mechanisms: thermal evaporation and explosive ejection process^24^. In the thermal evaporation process, the plasma plume and vapor are produced when a focused laser beam is allowed to interact on the surface of the target metal immersed in a liquid (solvent). The element of the target component can be either a single or mixed metal element, or a compound, based on the desired product. Then, the stronger confinement of the plasma plume including ablated materials expands into the liquid environment, which is associated with shockwave emission. Later, the plasma plume cools down during the expansion, and fast energy transfer to the surrounding liquid. This phenomenon produces a cavitation bubble, which then expands and collapses as the bubble extends its maximum radius within a hundred microseconds. During bubble collapse, NPs are released into the liquid environment to form a stable colloidal solution^18,25^. In the explosive ejection process, where solid fragments and nano- or microsized hot droplets are ejected directly from the target to form NPs. The laser parameters are mainly influenced by the dominating mechanism, i.e., plasma and vapor are generated (thermal evaporation process) when the high energy density of short-pulse lasers is used (nanosecond (ns) pulsed laser with several ns pulse width and power density of 10^8^–10^10^ W cm^−2^). Moreover, with the use of low-power density lasers (picoseconds (ps) or femtoseconds (fs) lasers), the fragments/nanodroplets (explosive ejection process) are obtained as the major product^24^. In the meantime, the nature of the solvent (type of solvent, temperature, and pH) can also affect NP formation.

During the PLI process, the formation of metal or alloy NPs via irradiation of nonfocused laser beams on the concerning metal salts as the precursor solution is ascribed to the optically induced decomposition of solvents without utilizing any other chemical reducing agents, which is called photoreduction. Upon laser irradiation in liquid (solvents: water or organic solvents), solvated electrons (e^−^_liquid_) and free radicals such as H^•^, and OH^•^ are generated (Eq. (1)), where e^−^_liquid_ and H^•^ act as reducing agents for the reduction of metal salts into corresponding metal NPs, as shown in Eq. (2)^1,11^.

1 $${{{\mathrm{H}}}}_{{{\mathrm{2}}}}{{{\mathrm{O + PLI}}}} \to {{{\mathrm{e}}}}^{{{\mathrm{ - }}}}{{{\mathrm{liquid + H}}}}^ \cdot {{{\mathrm{ + OH}}}}^ \cdot$$

In the case of organic solvents such as methanol and ethanol, CH_3_^•^ and CH_3_CH_2_^•^ radicals are generated.

2 $$\begin{array}{*{20}{l}}{{{\mathrm{M}}}}^{{{\mathrm{ + }}}}{{{\mathrm{ + e}}}}^{{{\mathrm{ - }}}}{{{\mathrm{liquid}}}}\,{{{\mathrm{and/or}}}}\,{{{\mathrm{H}}}}^ \cdot \to {{{\mathrm{M}}}}^0 \\ {{{\mathrm{where}}}}\,{{{\mathrm{M}}}}\,{{{\mathrm{is}}}}\,{{{\mathrm{the}}}}\,{{{\mathrm{targeted}}}}\,{{{\mathrm{metal}}}}\end{array}$$

**Fig. 2:**
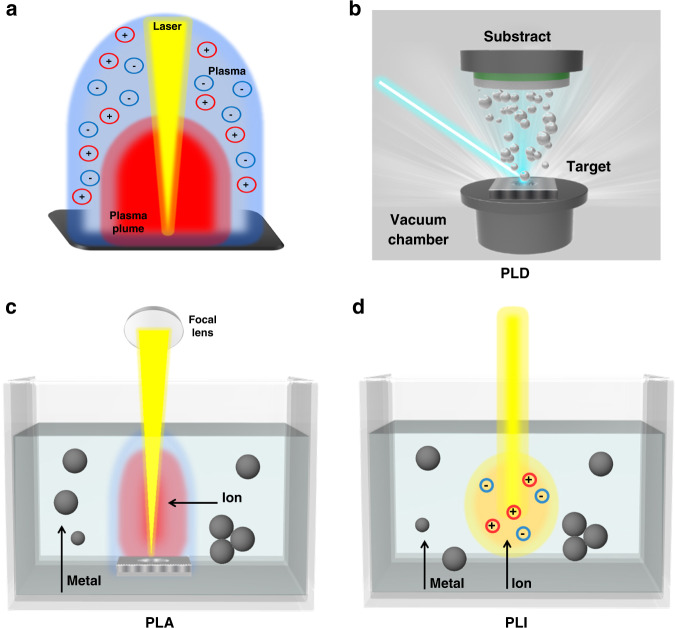
Schematic illustration of pulsed laser methods. **a** laser-induced plasma plume, **b** PLD, **c** PLAL, and **d** PLIL

If the experimental conditions are appropriately optimized, the PLAL and PLIL processes can produce uniformly distributed and highly stable NPs in a suitable solvent without adding any stabilizing/reducing agents, making them exceptionally advantageous for photo- and electrocatalytic applications. In general, the catalytic performance of nanomaterials strongly depends on their surface chemistry. Even though numerous nanostructured catalysts are attainable using conventional chemical synthesis routes, the catalyst surface is not optimal to enhance photo- and/or electrocatalytic performance or both because of the adsorbed reaction residues or stabilizing molecules, causing “barrier effect.” The ligand-free catalyst surface with high purity is highly advantageous for the interaction/reaction with adsorbents. The catalytic materials produced using the PLAL process are expected to show higher surface coverage than those produced using chemical routes, suggesting that materials with “clean and bare” surfaces are highly preferred for catalytic applications^3,24,26^. The schematic illustrations of the laser-induced plasma plume and PLD, PLAL, and PLIL processes are depicted in Fig. 2.

The selection of the appropriate laser system is crucial for the production of desired nanomaterials with controlled size and structure. The plasma plume and vaporization rate of the target source material are usually controlled by laser parameters (laser source/wavelength, fluence, pulse width, and pulse repetition rate/frequency), the condition of the liquid environment, and the efficiency of the light absorption behavior of target material.

### Laser source/wavelength

The laser wavelength is the main parameter that regulates the absorption efficiency of the fixed target, which can also influence the absorption depth and area of the focusing spot. In the early stage of the PLA synthesis of nanomaterials, an excimer laser in the UV region (ArF for 193 nm, and KrF for 248 nm) is used as a light source, which mostly employs gas as its active medium. With scientific advancements, the process utilized upgraded computer control, a sensor for monitoring the process, and scanning stage technology. Recently, Q-switch pumped pulsed Neodymium-doped yttrium aluminum garnet (Nd:YAG) laser, a solid-state crystal laser, has been commonly utilized for laser ablation since they do not involve hazardous gases. Nd:YAG lasers produce light at a *λ* of 1064 nm for the first harmonic wave (IR region), which can be altered by utilizing nonlinear optical crystals (e.g., second-harmonic laser *λ* at 532 nm and third harmonic laser *λ* at 355 nm).

**Fig. 3:**
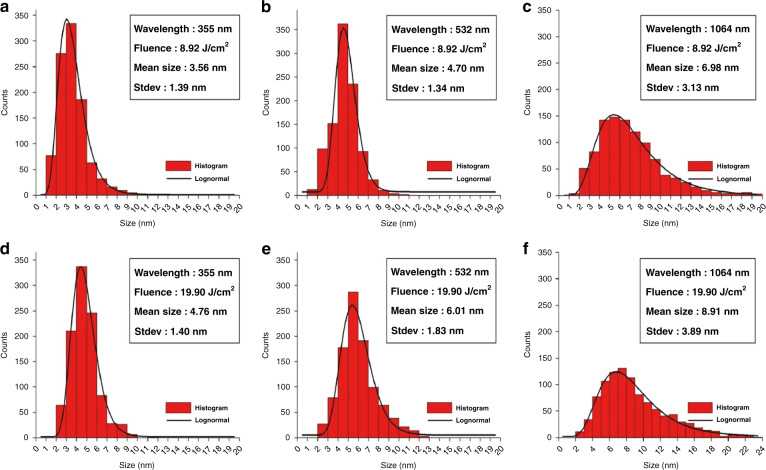
Average particle size distribution of Pd-NPs synthesized via the PLA of a Pd-foil in water at various laser wavelengths and fluences. **a**–**c** At fixed 8.92 J/cm^2^ fluence with various wavelengths, 355, 532, and 1064 nm, respectively, **d**–**f** at fixed 19.90 J/cm^2^ fluence with various wavelengths, 355, 532, and 1064 nm, respectively^28^. Copyright (2021) Elsevier

The laser wavelength determines the penetration depth (skin depth), and subsequently, the depth of ablation on the target. The penetration depth is higher for a higher wavelength of a pulsed laser, i.e., the penetration depth of a 1064-nm pulsed laser in the target is higher than that of the 532-nm pulsed laser (order of penetration depth can be followed as 355 nm < 532 nm < 1064 nm), which suggests that the ablated mass per pulse can increase with the increased pulsed laser wavelength. Furthermore, the laser wavelength strongly influences the produced amount and size of NPs in the PLAL process. In general, the amount (mass) of NPs is higher using a higher pulsed laser wavelength and subsequently, the NP size also increases at a higher wavelength^27^. Kim et al.^28^ studied the effect of various pulsed laser wavelengths (355, 532, and 1064 nm) and fluences (8.92 and 19.90 J cm^−2^) on the size and produced amount of Pd-NPs obtained by the PLA of a Pd target in water. As a result, the average particle size of Pd-NPs was increased with the wavelength at a fixed fluence: 3.56, 4.70, and 6.98 nm Pd-NPs were obtained by 355, 532, and 1064 nm laser wavelength at 19.90 J cm^−2^ fluence. The particle size increased along with the amount when the fluence increased at a fixed laser wavelength, as shown in Fig. 3^28^. At a lower fluence, the surface atom from the target is removed by evaporation, which leads to NP formation owing to the nucleation of evaporated atoms with uniform size distribution. Conversely, as the fluence increases, high energy is released to the target, which removes the material by melting. The interactions of metal droplets are fragmented by the laser beam and rapid quenching, causing the production of larger NPs with broader size distribution. Thus, the production of a large amount of Pd-NPs upon increasing the laser fluence occurred due to the ablation of the Pd target at high energy^28^.

### Pulse duration or pulse width

The laser pulse width, which is also called pulse duration, is also an important parameter in material synthesis. Varying it from ns to ps and fs changes the ablation mechanism from thermal evaporation to the explosive ejection process of chemical bond breaking at the target surface. A shorter pulse width results in the efficient ablation process, ensuring rapid evaporation and the smallest heat-affected region^29^.

### Fluence/energy density

The power of a laser is estimated in watts (W), which expresses either the average power of a pulsed laser or the optical output power of a continuous laser wave. Pulsed lasers are also considered by their pulse energy, which is inversely proportional to the laser’s repetition rate or directly proportional to the average power. Energy is estimated in joules (J).

Fluence (F) is defined as the laser energy per unit area (J cm^−2^) on the target source materials, expressed as F = *I*/A, where *I* refers to the laser power in J pulse^−1^, and A refers to the area of the laser spot on the target source in cm^2^. The PLAL process expanded to NP synthesis at various energy densities suggesting that the produced amount of materials increases with a higher energy density. Regarding particle size, the growth and nucleation theory can be employed to describe particle size variances based on energy density. The nucleation is challenging at a lower energy density, which leads to a small number of nuclei and bigger particle sizes, whereas nucleation takes place instantaneously in the case of higher energy densities, which leads to a large number of nuclei with smaller particle sizes^29^.

### Pulse repetition rate

The pulsed laser repetition rate is also known as pulsed repetition frequency, which is described as the number of output pulses emitted per second. The repetition rate is inversely proportional to the pulse energy. The impact of the repetition rate can be exactly determined only at fixed pulse energy. The cavitation bubble produced by the first pulse can shield the incident laser beam; in addition, the occurrence of an ablated particle in the path of the successive incident laser pulse reduces the maximum effectiveness of the repetition rate. Hence, some temporal repetition delay is required to permit the transmission of a consequent pulse to the surface of the target source without attenuation of an excessive intensity. Therefore, the optimization of the repetition rate is required at each intensity of the liquid–target source material system^27^.

Note that the shielding of ablation occurs when the second pulse attains during the first pulse cavitation produced; thus, the second bubble lifetime shortens due to the lower pulse energy in the reduced size of the cavitation bubble. The fluctuation in the cavitation bubble induced by the repetition rate remains there until it reaches the maximum steady state for several pulses^27^. At the point when the repetition rate is about ≤1 kHz, the produced NP amount increases with the increased repetition rate, even at high pulse energies. However, more sophisticated laser scanners are needed for higher repetition rates^27^.

### Laser-focusing parameters

**Fig. 4:**
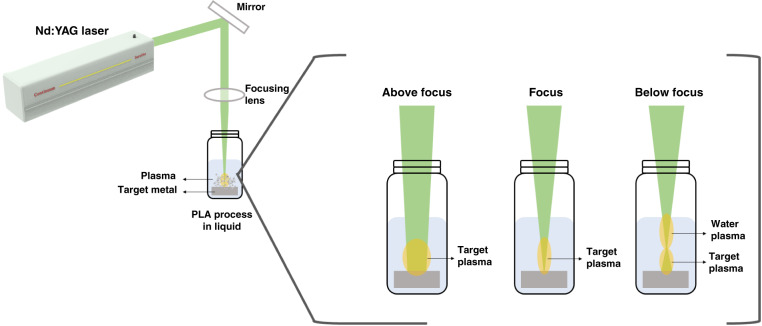
Schematic illustration view of the PLA process in liquid medium with various target positions: above-focus, at-focus, and below-focus conditions

The condition of the laser focusing point (target position) is a crucial parameter, which influences the size distribution and shape of the NPs. The ablation of the target position can be changed by altering the position of the lens using a micrometer screw, which is attached to the mount of the lens. At a fixed target, the ablation can be performed at three positions (above-focus or defocus, at-focus or focal point, and below focus), as illustrated in Fig. 4.

**Fig. 5:**
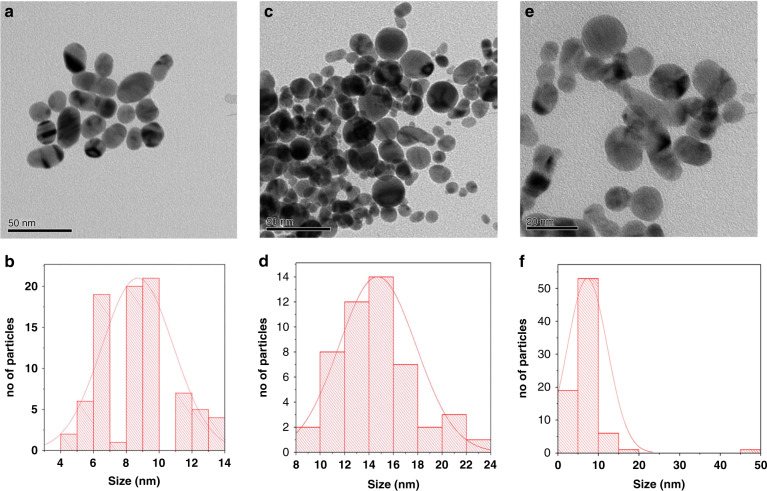
TEM images and histograms of Au-NPs produced by ablation in water at various target positions. **a**, **b** above-focus, **c**, **d** at-focus, and **e**, **f** below-focus conditions^30^. Copyright (2021) Scientific Research Publishing

The average particle size is larger when the target is ablated at the focal point compared to the above-focus and below-focus conditions, while smaller particle size is obtained at the below-focus condition^30,31^. A detailed study about the effect of target position on the particle size of Au-NPs via PLA was reported by Imam et al.^30^. During the PLA of the Au plate in water at the above-focus point, an average particle size of around 8.6 nm was obtained with a low yield, which might be due to the low fluence, and breakdown of water (or any solvent) medium does not occur at the above-focus point. When the target is heated at this fluence, the vaporization rate is limited owing to the strong liquid confinement on the target surface. The resulting TEM image and histogram of Au-NPs synthesized by ablation at the above-focus point are shown in Fig. 5a, b. The Au-NPs obtained at the focus condition show an average size of about 8.9 nm with a spherical shape, as depicted in Fig. 5c, d, which is larger than the Au-NPs obtained at the above-focus (defocus) condition. The PLA at the focus point follows the thermal evaporation mechanism, as explained in the section “Fundamentals of the pulsed laser process”. When the target was ablated at the focus point, the temperature, plasma pressure, and density increased compared to those at the defocus condition. The high plasma temperature excites, ionizes, and the surrounding water medium dissociates, initiating the nucleation directly. The increased particle size might be due to the enhanced particle growth and molecular integration by the high plasma density. On the contrary, Au-NPs obtained by below-focus ablation shows a smaller average particle size (~7.2 nm) than those produced at the defocus and focus conditions, as shown in Fig. 5e, f, likely because when the target was ablated at the below-focus condition, the maximum amount of energy was absorbed by the liquid (water) medium, reducing the energy that reaches the target. Another investigation by Nath et al.^31^ also confirmed this explanation, wherein, TiO_2_ NPs were prepared via the PLA of the titanium target plate in water at various target positions, and the average size of TiO_2_ NPs prepared at the focus point was higher than those produced at the defocus (above-focus) and below-focus conditions. The additional information about spot size was also provided, which mainly depends on the target position. The minimum spot size is preferred for effective NP production. The spot size is smaller when the target is ablated at the focus point, while it is larger at the above-focus condition, as shown in Table 1.

**Table 1:** Effects of laser and NPs parameters during the ablation of the Ti target in a water medium in a fixed focal length of 5 cm at various target positions^31^

Target position	Spot size (μm)	Fluence (J cm^−2^)	Average particle sizes (nm)	Particle size distribution (nm)
Above focus	~600	1.4	12	2–45
Focus	80	80	13	4–205
Below focus	400	0.2	9	3–75

Copyright (2021) Elsevier

### Solvent medium

The solvent medium is another important source in PLAL, which can control not only the shape and size of NPs but also the structure and composition of the final product. A detailed explanation of the solvent effect is provided in this section.

#### Effect on crystal structure

**Fig. 6:**
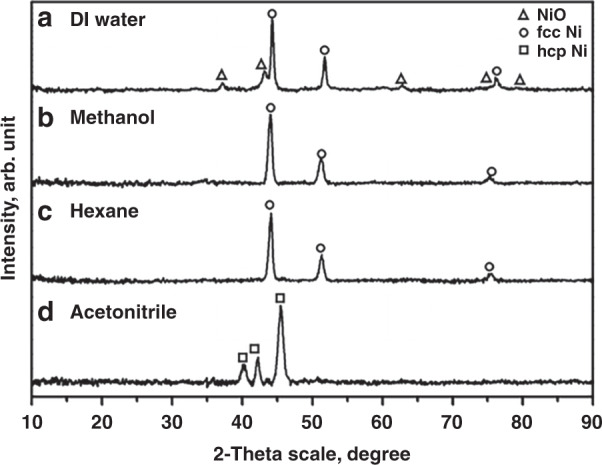
XRD patterns of NPs prepared using the secondary irradiation of the obtained different phases of Ni NPs via the primary ablation of the Ni plate using various solvents. **a** DI water, **b** methanol, **c** hexane, and **d** acetonitrile^32^. Copyright (2021) American Chemical Society

Our research group investigated the effect of various solvents [DI water, methanol, hexane, and acetonitrile (ACN)] on the production of Ni NPs by PLA using a 1064-nm Nd:YAG laser with a focal length of 30 mm, and the PLA reaction was performed for 20 min^32^. Interestingly, it was identified that the specific solvents resulted in specific Ni NP phases; fcc Ni/NiO and pure fcc Ni NPs were obtained in DI water and methanol medium, respectively, while a mixture of fcc/hcp Ni phases was obtained in both hexane and ACN. The observed results indicated that NP formation during the PLA process is strongly dependent on the solvent medium; as a result, the specific heats of different solvents produce the distinct phases of Ni NPs. We further performed secondary laser irradiation for the obtained colloidal solution for another 20 min and noticed that there were no changes in DI water and methanol samples; however, pure fcc Ni and hcp Ni phases were obtained in hexane and ACN, respectively, and the resulting XRD pattern is shown in Fig. 6. This result can be explained as the phenomena of plasma plume cooling experienced by Ni NPs, which differ significantly for various solvents, leading to different Ni NP phases. During adiabatic expansion and plasma plume cooling, specific heats of the solvents can contribute to nuclei growth and nanocrystal coalescence. Particularly, the high pressure and temperature of the plasma plume induced by the laser are uniquely produced at the interface of the liquid–solid target. Then, the plasma plume condensates at various cooling rates in different confining solvents due to the liquid confinement effect to produce Ni NPs with various phases. Considering the high-temperature and high-pressure region on their thermodynamic phase equilibrium diagram, a high temperature of ~6000 K and high pressure of ~10 GPa are favorable for the production of the metastable phases. In this case, the high cooling rate of the plasma plume in the confined liquid enhances the metastable NPs. Thus, the cooling rate mainly depends on the specific heats of solvents; those with lower specific heat cool the plasma plume efficiently and produce the metastable phase of hcp Ni NPs (specific heats: ACN (2.2 J kg^−1^) < hexane (2.3 J kg^−1^) < methanol (2.5 J kg^−1^) < DI water (4.2 J kg^−1^)). Furthermore, the NP size is reduced upon second irradiation, resulting in the fragmentation of the formed NPs in the primary ablation and renucleation into smaller sizes. Thus, the additional laser irradiation selectively heats and vaporizes bigger particles, and the consequent rapid cooling of the vaporized particles initiates the production of smaller NPs.

**Fig. 7:**
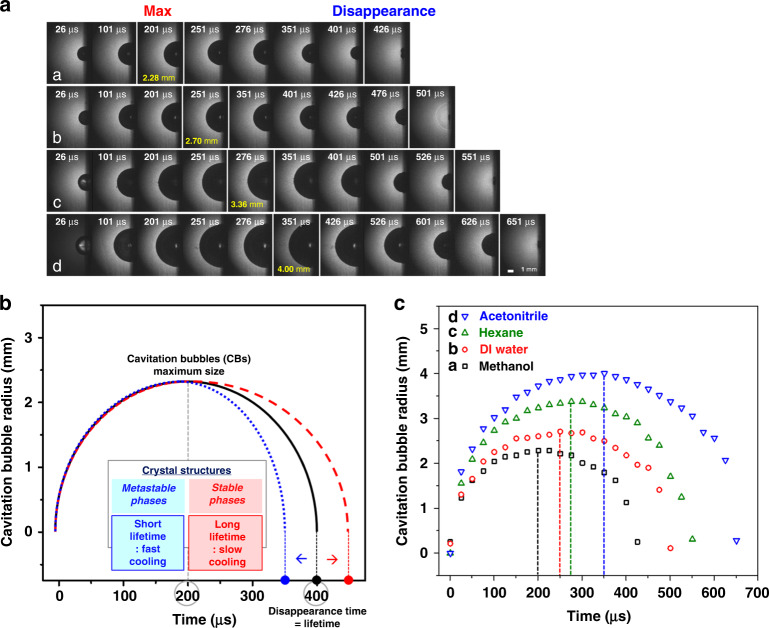
Dynamics studies of LICBs. **a** Time-resolved images of LICBs taken during the PLA of the Ni plate for 30 min in (a) methanol, (b) DI water, (c) hexane, and (d) ACN medium; **b** theoretical lifetime values of LICBs obtained based on the Rayleigh–Plesset theory; **c** time-resolved LICB radius evolved during the PLA of the Ni plate in (a) methanol, (b) DI water, (c) hexane, and (d) ACN medium^25^. Copyright (2021) Elsevier

The above investigation was further confirmed by our research group with time-resolved dynamics studies of laser-induced cavitation bubbles (LICBs) during Ni NP formation via PLA in various solvents^25^. Herein, the time-resolved formation of LICBs was measured using an intensified charge-coupled device camera, and it was found that the lifetime of LICBs is dependent on the solvent medium and that the phase of Ni NPs is defined by the LICB lifetime during the PLA process. Especially, the short lifetime of LICBs in ACN leads to the formation of metastable hcp phase Ni NPs, while the long LICB lifetime produced a stable fcc Ni phase in methanol. In detail, the formation of Ni NPs takes place via the plasma plume at high pressure and temperature during PLAL. The phase of Ni NPs is altered according to the plasma plume cooling rate. When the cooling rate is slow, the lifetime of LICBs is long; thus, Ni NPs have enough time to produce thermodynamically stable fcc structures. On the contrary, when the cooling rate of the plasma plume is rapid, the lifetime of LICBs is short, and Ni NPs have a metastable hcp phase. The study of the dynamics of LICBs showed the mainly solvent-dependent lifetime and size of LICBs. The time-resolved images of the LICBs taken during the PLA of the Ni plate for 30 min at various solvents are shown in Fig. 7a; the maximum size of LICBs (2.28, 2.70, 3.36, and 4.0 mm) reached at 201 μs (methanol), 251 μs (DI water), 276 μs (hexane), and 351 μs (ACN), respectively. Consequently, the LICBs began to collapse and disappeared completely at 426, 501, 551, and 651 μs, respectively (Fig. 7a, c), as presented in Table 2. Figure 7b represents the theoretical lifetime value of LICBs based on the Rayleigh–Plesset theory. If they had collapsed before the theoretical lifetime value, the LICBs would have revealed a shorter lifetime, but they disappeared after the theoretical lifetime value, exhibiting a longer lifetime. Herein, the LICBs produced in the ACN medium showed a shorter lifetime, while the LICBs produced in methanol exhibited a larger longer lifetime. As a result, it is confirmed that the lifetime and size of the LICBs highly depend on the solvents used in the PLA process^25^.

**Table 2:** Time-evolution dynamics of LICB lifetime obtained for the PLA of the Ni plate for 30 min in various solvents^25^

Solvent	*T*_max_, μs	Collapse time, μs		Lifetime (*T*_the_:*T*_exp)_
		*T*_theoretical_	*T*_experimental_	
Methanol	201	402	426	<, long
DI water	251	502	501	=, equal
Hexane	276	552	551	=, equal
Acetonitrile	351	702	651	>, short

Copyright (2021) Elsevier

#### Effect on composition and morphology

**Fig. 8:**
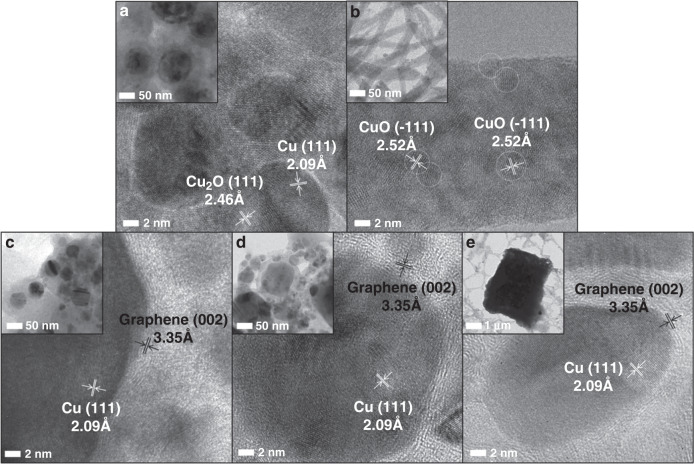
HR-TEM surface morphology with lattice distances of materials obtained by the PLA of the Cu-plate in various solvents. **a** DI water, **b** methanol, **c** ethanol, **d** hexane, and **e** ACN. The insets show the TEM images of the resulting products^10^. Copyright (2021) Elsevier

Recently, our research group has investigated the effects of various solvents (DI water, methanol, ethanol, 1-propanol, butanol, ethylene glycol, hexane, and ACN) on the final composition and surface morphologies of Cu-NPs synthesized via the PLA of the Cu target using 1064-nm Nd:YAG (laser energy: 80 mJ pulse^−1^) for 10 min^10^. Interestingly, the PLA of the Cu-plate in ethanol, 1-propanol, butanol, and ethylene glycol provided fcc Cu-NPs with a spherical shape, whereas ACN leads to CuCN cubes. The ablation of the Cu-plate in DI water and methanol leads to the formation of Cu_2_O and CuO with spherical and rod-like structures, respectively, and corresponding high-resolution transmission electron microscopy (HR-TEM) images with lattice distances are depicted in Fig. 8. During the ablation of the Cu target, the high temperature and pressure of the plasma plume lead to the decomposition of solvent molecules, producing O_2_, H_2_, free radicals, and carbon-based fragments. Subsequently, the vaporized metal ions condensed and reacted with these species to produce the resulting materials. Thus, the formation of different compositions and morphologies of Cu-based NPs is due to the bond energy differences in various solvents, which play a vital role in solvent decomposition and affects the final products^10^.

Methanol can release the OH group by bond breaking between C and O atoms and thus lead to the formation of Cu(OH)_2_. Then, the decomposition can occur during the PLAL process and form CuO rods. In the case of carbon-rich solvents (ethanol, 1-propanol, butanol, hexane, and ethylene glycol), the binding energy of C1–C2 is lower, which is more favorable to dissociate than that of the C–O bond. As a result, carbon atoms decomposed from the carbon-rich solvents are deposited as the GC layer on the Cu NP surfaces (a more detailed mechanism about carbon layer formation is provided in the section “Solvent as an element source”). The bond length of these solvents is also consistent with the bond breakage orders, where the C–C bond length is longer than that of C–O; hence, the dissociation of the C–C bond can occur earlier than that of the C–O bond. Therefore, the formation of CuO is less favorable in the case of long-carbon-chain solvents. On the other hand, a new phenomenon was observed during the PLA of the Cu-plate in ACN, which led to the formation of CuCN as the predominant product. Thus, the formation of CuCN is due to the generation of the cyano group by the decomposition of ACN solvents, which can form strong complexation with metal ions to form CuCN^10^.

**Fig. 9:**
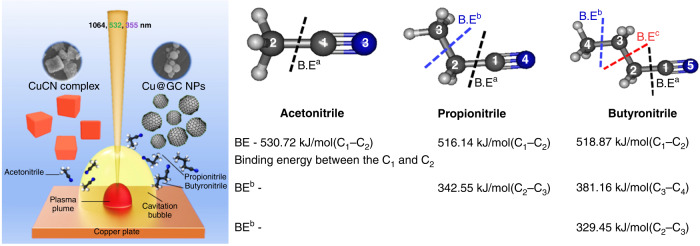
Schematic illustration of the PLA of the Cu-plate in various nitrile solvents, and the structure of organic nitrile solvents with calculated binding energies^33^. Copyright (2021) Springer Nature

Further investigation on the formation mechanism of CuCN was conducted by the ablation of the Cu-plate in various nitrile solvents with different binding energies (acetonitrile, propionitrile, and butyronitrile)^33^. The cube-like CuCN was formed by the reaction of oxidized Cu ions with CN^−^ groups obtained by the dissociation of ACN, whereas other nitriles (propionitrile and butyronitrile) produced spherical graphitic-carbon-covered Cu-NPs (Cu@GC NPs). Thus, the formation of CuCN occurs in ACN only due to the easy accessibility of the CN^−^ group after the dissociation of ACN into CH_3_^+^ and CN^−^ by PLA, whereas propionitrile and butyronitrile solvents generate hydrocarbons in large amounts, which can be deposited as GC layers on the surface of Cu-NPs and hinder the interaction of the CN^−^ group with Cu-NPs. The formation mechanism was also confirmed by the theoretical quantum chemical calculations on the binding energy of various nitrile solvents, as presented in Fig. 9^33^.

#### Solvent as an element source

Its commonly known that the free radicals are produced by the decomposition of solvents during the laser irradiation/ablation process, which can act as reducing agents. Also, carbon-rich solvents can be decomposed during laser ablation and condensed as a carbon layer on the NPs. More recently, our group utilized a solvent (DMSO) as a sulfur source for the preparation of ZnS^34^. In detail, ZnS nanospheres were synthesized by the PLA of the Zn plate in DMSO without using any additional sulfur source, reducing agents, or surfactants. DMSO acted as both a solvent and sulfur source. During the ablation of the Zn plate, Zn^2+^ ions are generated and vaporized along with the plasma plume and subsequently reacted with the sulfur source obtained by the decomposition of DMSO to form ZnS. Similarly, others have reported the utilization of DMSO as a sulfur source for the one-spot production of various metal sulfides, including cadmium sulfide (CdS), CuS, and tin sulfide (SnS)^35,36^.

**Fig. 10:**
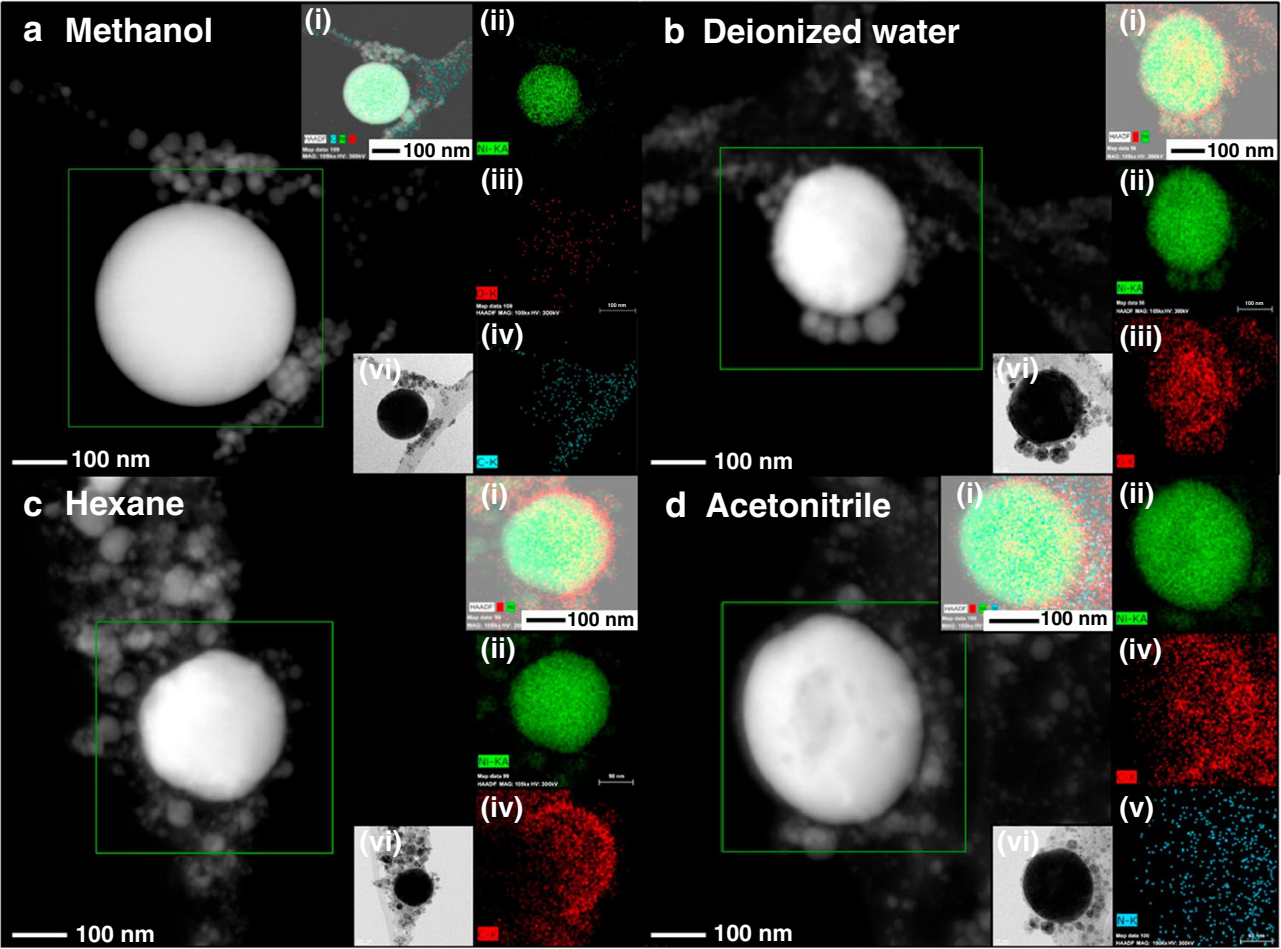
Elemental mapping images of materials produced by the ablation of the Ni plate in various solvents. **a** methanol, **b** DI water, **c** hexane, and **d** ACN. The insets show the images of the **i** overall, **ii** Ni, **iii** O, **iv** C, **v** N, and **vi** resulting TEM images^37^. Copyright (2021) Elsevier

Another report by our research group utilized ACN as a carbon and nitrogen source for the production of N-doped GC-covered Ni NPs (Ni@NGC NPs)^37^. The Ni target was ablated in various solvents, such as DI water, methanol, hexane, and ACN. As a result, Ni@NiO and Ni NPs were produced in DI water and methanol medium, respectively, whereas GC and Ni@NGC NPs were produced by hexane and ACN, and the corresponding TEM and elemental mapping images are shown in Fig. 10.

During the adiabatic cooling of Ni NPs, the H, C, and N atoms or ions, and CH_3_^+^ and CN^−^ ions obtained by the decomposition of the solvents (hexane and ACN) are condensed on the surface of Ni NPs and produce the GC- and NGC-covered Ni NPs^37^. However, the doping of N is not favorable in all metal NPs, which is strongly based on the solubility of N on the metal. For example, a similar investigation was conducted using Au-NPs, and both hexane and ACN produced the GC layer on the Au-NPs. Thus, the formation of Au@GC NPs rather than Au@NGC NPs in ACN may be ascribed to the fact that the energy formation among the Au atomic lattice is ~5 times higher than that of Ni. The formation energy of N in Au lowers the solubility of N in Au-NPs, making N doping in the GC layer difficult^38^. It can be suggested that alloy or bimetallic NPs with appropriate solvents such as N or C sources can be obtained by tailoring Metal@NGC or Metal@GC NPs.

The next section describes the key works related to material production via pulsed laser-assisted techniques, especially PLA and PLI processes in liquid media. The effects of laser parameters, such as laser fluency (time duration, pulse width, wavelength, and repetition rate), focusing parameters (target position and focal length), and the liquid medium (type of solvent, plasma plume) on the formation of diverse functionalized nanostructures are discussed.

## Photo- and electrocatalytic materials: an overview

### PLAL process

In the past decades, the progress of human civilization has been completely centered on fossil fuel exploitation, which has generated substantial crises, such as environmental pollution and global warming^39,40^. Owing to the sudden growth of the human population, there was an increasing shortage of fossil fuels. To resolve this, scientific community has proposed to upgrade the energy sectors with wide plausible technologies; however, only moderate progress has been made owing to economic deliberations. Therefore, developing inexpensive, clean, green, and renewable energy sources, although challenging, is crucial. In the meantime, the evolution of energy materials equals pivotal, which helps the conversion, storage, and harvest of clean sources. Pulsed laser technology has been vastly used in time-resolved characterization techniques, including fluorescence or transient-absorption spectra, which simplify the progress of basic science. Moreover, in nanomaterial synthesis, the pulsed laser process has been espoused largely in a technology labeled as the PLA. Recently, PLAL has received great interest in photo/electrocatalytic process owing to its several advantages, such as facile experimental setup, ecofriendliness, long-term nanoparticle stability, and lack of undesired impurities or toxic precursors. PLAL is used to fabricate numerous nano- and submicron-sized materials with unique topographies, phases, and structures via a single-step process, realizing their applications in sensors, displays, catalysts, optics, and biological fields. Though numerous works were reported in PLA, the acceptance of this technique in catalysis-oriented applications is relatively undervalued.

Generally, the surface chemistry of the NPs is one of the decisive factors to determine their catalytic activity. Though chemical synthesis was largely applied to achieve metal nanostructures, the NP’s surface is not ideal for the catalytic process owing to firmly adsorbed reaction residues or surface-stabilizing compounds, hindering the catalysis and poisoning the NP catalysts. For such requirements, catalysts with ligand-free surfaces and high purity are highly essential to interact with their adsorbents. In this regard, catalytic NPs obtained using PLAL are shown to retain a five-fold higher surface area coverage than their chemical process counterparts suggesting improved efficiency for the surface interaction with the adsorbents. Thus, metal nanostructures derived using PLAL with clean and bare surfaces are highly desired for catalysis, including photo- and electrocatalytic methods. In this section, the key properties and functions of PLAL-based fabrication of photo- and electrocatalytic nanomaterials, including MNPs, core-shell NPs, oxides, and nonoxide nanomaterials with unique structures, and their salient features are discussed.

#### Metal nanoparticles

In general, MNPs possess a wide range of specific (optical, electrical, and magnetic) properties compared to their bulk counterparts. These specific properties largely depend on their particle size, distribution, surface structure, and composition. For instance, Si-NPs are highly active in the visible region and photoluminescent at room temperature due to their particle size, which could be used to control the wavelength of emitted light. Interestingly, when their particle size is at the nanometer scale, MNPs possessed unique characteristics, including fluorescence, paramagnetism, ferromagnetism, and spin quantum effect. As mentioned earlier, bottom-up liquid-phase methods (chemical reduction and sol–gel) are used to produce MNPs with well-defined specific nanostructures, whereas bottom-up vapor phase (CVD, PVD) approaches yield high-purity MNPs. Both bottom-up approaches (liquid and gas phases) provide powder MNPs derived from the nucleation of super-saturated species as the end products, and both processes have their own advantages and disadvantages. Henceforth, to fabricate high-purity MNPs with well-defined nanostructure using a cost-effective method, Neddersen et al. introduced the PLA method by ablating the solid-target material by means of a laser energy source^41^.

Based on the PLA process, numerous works have been devoted to fabricating MNPs in the early 90s. The Au and permalloy (80% Ni–20% Fe) microspheres with diameters of 2 and 1–10 μm, respectively, under ambient conditions, have been generated by Cai et al., which provided a very small mean diameter and good dispersion compared to other conventional flat-surface methods^42^.

In the upcoming subsections, we focus on the properties, function, and recent advancement of PLAL (ablation in liquids)/PLIL (irradiation in liquid) related metallic NPs in photo- and electrocatalytic processes. We start with the representative examples of the PLAL-based metallic NPs, oxides, and their composites for photo/electrocatalytic processes and then move out to deliver the salient features of PLIL for various catalytic processes. Furthermore, recent developments about using PLAL- and PLIL-driven metal NPs are briefly summarized.

#### Noble metals

Colloidal noble MNPs are of huge importance for applications in a broad range of disciplines because of their unique optical and electrical properties. Different laser sources and solvents have been reported to synthesize various morphologies of Ag and Au-based nanostructures^43,44^. For example, Simakin et al. developed nanodisk-shaped Au and Ag with an average diameter of 20–60 nm through the PLAL process without including any counter ions and surface-active agents^45^. For this process, a Cu-vapor laser was used as an energy source, which ablated Ag and Au targets in water and produced their respective colloidal sols. These Ag and Au-based aqueous sols were further confirmed by the presence of plasmon bands at 400 and 520 nm through UV-vis spectroscopy. Using an Nd:YAG laser source with *λ* = 1064 nm, a similar type of Ag-NPs was generated by PLAL in different concentrations of NaCl and clear water solutions by Bae et al.^46^. Compared to other concentrations, 5 mM NaCl led to a particle size between 5 and 50 nm with an average particle diameter of 26 nm. Thus, the presence of Cl^−^ ions in H_2_O reduced the particle size of NPs, which resulted in improved NP formation during the PLAL process. Ag-NPs fragmented by the absorption of the laser light at 355 nm in 5 mM NaCl solution have been observed to be highly effective. Interestingly, when Ag-NPs were fabricated in acetone at different irradiation times and laser wavelengths (266, 400, 532, and 800 nm), they demonstrated different morphologies ranging from spherical to nanowire. The mechanism for Ag nanowire formation depends on the type of laser light used and the irradiation time. The achieved nanowires had an average size and length of 100 and 600 nm, respectively^47^. Based on these findings, Fazio et al. developed both solid and colloidal Ag and Au-NPs through the PLA process in water to improve the surface-enhanced Raman scattering (SERS) of conventionally available red organic dyes, such as carmine, bazilwood, madder lake, and dragon’s blood, using an Nd:YAG laser at 532 nm under inert atmosphere^48^. Using different laser times such as 6 ns, 200 ps, and 60 fs, Ag-NPs with 8 nm have been generated by Boltaev et al. in water and air media^49^. Their optical properties were studied at two wavelengths and observed the nonlinear absorption coefficient of 3 × 10^−5^ W cm^−1^. Vinod et al. synthesized monodispersed Au:Ag-NPs by PLAL for the improved SERS activity. They used an Nd:YAG energy source at two wavelengths (355 and 1064 nm) for ablating target materials^50^. The highest SERS activity with an enhancement order of about 10^5^ has been achieved for <Au_80_:Ag_20_>_1064_.

**Fig. 11:**
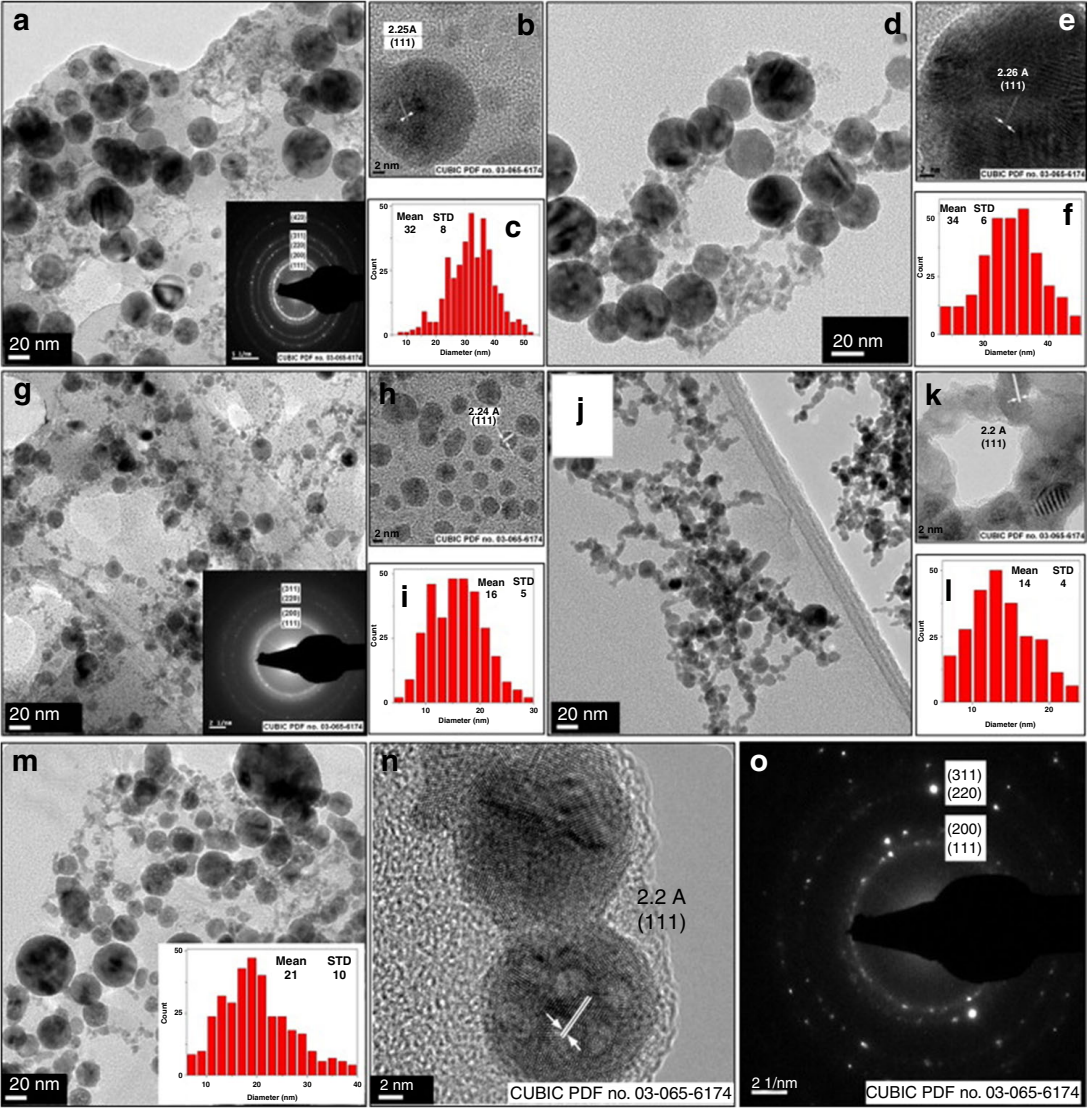
HR-TEM images of PLAL-synthesized Pd-NPs after post-laser irradiation and sonication. **a**–**f** DI water, **g**–**l** methanol–DI water mixture, **m**–**o** 1 mM SDS. The insets represent their corresponding particle size distributions^54^. Copyright (2021) Elsevier

Moreover, Pd and Pt are widely employed as automotive and industrial catalysts due to their potential advantages, including selectivity, lifetime, reusability, and activity, especially in energy and environmental areas. When these metals are in colloidal NP form, they can adsorb a huge quantity of ligands because of their large surface area. Like other MNPs, the formation of colloidal Pd-NPs with unique nanostructures depends on various parameters, such as solvents, laser fluence, laser wavelength, surfactants, and irradiation time^28^. Cristoforetti et al. investigated the effect of surfactant (sodium dodecylsulfate) on the production of Pd-NPs by PLAL using various solvents (ethanol, 2-propanol, acetone, toluene, and n-hexane)^51^. As a result, Pd-NPs possessed appreciable SERS activity and adsorbed large organic molecules. Nishi et al. testified the behaviors of Pd-NPs obtained by the ablation of the Pd target in the light and heavy water samples^52^. The average diameter of Pd-NPs produced in the heavy water was much smaller than that of Pd-NPs produced in the light water. Based on the M–H curves, they concluded that the magnetic susceptibility of Pd-NPs produced in the heavy water was larger than that produced in the light water and commercial Pd-NPs. Bonis et al. investigated the dynamics of multiple cavitation bubbles created by femtosecond PLAL of Pd in acetone through a rapid time-resolved shadow-graph process and observed that the size distribution of the laser-induced Pd-NPs is highly related to cavitation bubble dynamics^53^. The solvent effect using the Pd target in water and methanol/water mixtures using SDS as a surfactant has recently been identified through the PLAL process^54^. In this process, the spherical Pd-NPs with an average diameter of 27–13 nm have been generated using the Nd:YAG laser with a wavelength of 1032 nm and energy fluence of 40.5–8 J cm^−2^. Based on the TEM results as shown in Fig. 11, it was observed that the NPs aggregated more in water than in the methanol/water mixture. Also, SDS facilitated the particle size and agglomeration control (Fig. 11). In addition, laser post-irradiation and ultrasonication processes have been employed to redisperse Pd NP precipitates, which greatly enhanced the optical properties. Recently, Au, Pt, and Pd-NPs have been synthesized through the PLAL process in water, demonstrating excellent catalytic behavior under ambient conditions^24^.

Pt and its alloy nanostructures are gaining interest owing to their plausible applications in many catalysis reactions. Between various synthesis routes including hydrogen reduction, radiolysis, and citrate reduction, PLA is one of the cost-effective, rapid, versatile, and green routes to generate Pt and Pt-based compounds at the nanometer scale. Recently, the PLAL syntheses of Pt-NPs with different shapes, sizes, and properties have been reported by different research groups^55,56^. For instance, Mafune et al. developed stable Pt-NPs with the range of 1–7 nm in size distribution through PLA in water using SDS as a surfactant^57^. In this process, the formation of Pt-NPs in SDS was related to the dynamic formation mechanism^58,59^. In brief, the laser ablation of the Pt-target in SDS generates the clouds of Pt atoms over the laser spot of the Pt plate surface, which aggregate quickly into small particles of Pt. This process occurs until available Pt atoms are present in the closest vicinity and create more embryonic particles with a diameter of ~2 nm. These embryonic Pt-NPs grow by the addition of Pt atoms and diffuse into the void region after the rapid-growth process stops. Similarly, Yan et al. produced hollow micro/nano-Pt particles by the ablation of the Pt-target in water using different laser fluences (2.3–6.8 J cm^−2^)^60^. Other colloidal transition MNPs of inexpensive noble metal Ru have also been fabricated by the PLAL method^61^. Non-noble colloidal MNPs such as Si, Ni, Al, and Zn have also been synthesized through the PLAL process in liquid, as presented in Table 3^62–65^.

**Table 3:** Various laser parameters, including laser source, fluences, irradiation time, and solvents of PLA-processed metal and alloy nanoparticles

No.	Metal targets	Solvent	Products	Laser source	Condition	Particle size (nm)	Morphology	Ref.
1	Ag and Au-plates	Water	Ag and Au sols	Cu-vapor	*λ* = 510.6 nm; 32 J cm^−2^ for Au-NPs; 30 J cm^−2^ for Ag-NPs	20–60	Disk shape	^45^
2	Ag-metal	Aqueous solution of NaCl	Ag-NPs	Nd:YAG	*λ* = 1064 nm; 5 ns; 10 Hz; focal length 200 mm;	5–50	NPs	^46^
3	Au-metal	Water	Au-NPs	KrF excime	*λ* = 248 nm; 25 ns; 10 Hz; 2 J cm^−2^	–	–	^48^
	Ag-rod		Ag-NPs	Nd:YAG	*λ* = 532 nm; 5 ns; 10 Hz; 1.5 J cm^−2^	–	–	
4	Ag and Au-target	Water	Ag-NPs			16–26		^50^
			Au-NPs	Q-switched Nd:YAG	*λ* = 355 nm; 6–7 ns; 10 Hz; 11.94 for Ag J cm^−2^; 17.9 J cm^−2^ for Au	12–20	Spherical	
			Au_20_:Ag_80_			–		
5	Pd-foil	Ethanol–water	Pd-NPs	Nd:YAG	*λ* = 355, 532, and 1064 nm; 3–6 ns; 10 Hz; 8.92, 12.74, and 19.90 J cm^−2^	3–6	NPs	^28^
6	Pd-plate	Aqueous solution of SDS	Pd-NPs	Nd:YAG	*λ* = 1064 nm; 12 ns; 10 Hz; 1.6–2000 J cm^−2^	4.5–12.3	Spheroidal NPs	^51^
7	Pd-metal	Water; water–methanol mixture (1:1); SDS as surfactant	Pd-NPs	Nd:YAG	*λ* = 1064 nm; 10 ns; 10 Hz; 40.5–8 J cm^−2^	17 ± 6, 24 ± 7, and 27 ± 9 for different fluences	Spherical NPs	^54^
8	Pt-target	Ethanol–TSC solution	Pt-NPs	Nd:YAG	*λ* = 1064 nm; 10 ns; 10 Hz; 40.5–8 J cm^−2^	7–9 nm; 10–12 nm	Spherical NPs	^55^
9	Pt-metal plate	Aqueous solution of SDS	Pt-NPs	Q-switched Nd:YAG	*λ* = 532 and 1064 nm; 10 ns; 10 Hz; 1.6 J cm^−2^	1–7 nm	Nanoparticles	^57^
10	Silicon wafer	Ethanol–water	Si-NPs	Nd:YAG	*λ* = 1064 nm; 10 ns; 10 Hz; 50–200 mJ pulse^−1^	6.8 nm for 50 and 3.1 nm for 200 mJ pulse^−1^	Spherical at low fluence; Agglomerated NPs at high fluence	^58^
11	Zn-foil	THF	Zn-NPs	Picosecond laser system	*λ* = 515 nm; 10 ns; 10 Hz; 125 μJ; focal area 3.75 m/s	4.5 nm	Nanoparticles	^62^
12	Silicon wafer	NA	Si-NPs	Nd:Y: Al: garnet laser	*λ* = 532 nm; 10 ns; 10 Hz; 200 mJ; under He flow in a ablation chamber ~900 ^o^C	3–6 nm	NPs	^65^
13	AuAg-solid foils	Methyl methacrylate	AuAg-alloy	Picosecond pulse laser system	*λ* = 515 nm; 7 ns; 33.3 Hz; 125 μJ; focal length 56 mm	5–15 nm	Clusters of nanoparticles	^71^
14	AuAg-targets	Acetone solution of HAuCl_4_ and AgNO_3_	Ag_0.65_Au_0.35_ Ag_0.5_Au_0.5_ Ag_0.35_Au_0.65_	Femtosecond laser pulse	*λ* = 532 nm; 40 fs; 10 Hz; 150 μJ; 40 min; focal length 3 mm	≥20 nm	Spherical nanoparticles	^72^
15	Ag-foil, Au-foil	Water	Ag_100-*x*_Au_*X*_ bulk alloy	Nd:YAG ns-laser	*λ* = 532, 1064 nm; 60 ns; 100 Hz; 575 mJ cm^−2^;	10 nm	Spherical colloidal particles	^73^
16	Au_73_Fe_27_ metal plate	Ethanol	AuFe-alloy	Nd:YAG Quantel YG981E-laser	*λ* = 1064 nm; 9 ns; 10 Hz; 30 J cm^−2^;	30–60 nm	Crystalline nanoparticles	^74^
17	Au-plates; raw Fe_2_O_3_ nanoparticles	Ethanol	AuFe-alloy	Nd:YAG -laser	*λ* = 355 nm; 7 ns; 10 Hz; 150 mJ cm^−2^;	400–500 nm	Sub-micrometer Spherical nanoparticles	^75^
18	Pt_9_Ir target	Acetone	PtIr-alloy	Femtosecond laser	*λ* = 800 nm; 120 fs; 5 Hz; 300 μJ; focal length 150 mm	10–66 nm	Spherical nanoparticles	^77^
19	Ni_55.49_Ti_44.49_-target	water	NiTi-alloy	Femtosecond laser	*λ* = 1030 nm; 300 fs; 200 kHz; 8.5 μJ; focal length 3 mm	100–200 nm	Nanoparticles	^79^
20	AlCuFe QC alloy target	Ethanol; water	AlCuFe-alloy	Nanosecond Nd:YAG laser	*λ* = 1064 nm; 8 ns; 10 Hz; 40, 80 J cm^−2^; focal length 6 mm	3–37 nm	Spherical nanoparticles	^80^
21	Bulk Al-surface coated with Au-film	Ethanol	Au@Al_2_O_3_ core-shell	Continuous wave fiber laser	*λ* = 1064 nm; laser power 40 W; thickness 1 mm; focal length 43 cm	10–18 nm	Conical cavities of spherical particles	^81^
22	Fe-sheet	Acetone	Fe@C; Fe@FeO_*x*_	Femtosecond laser	*λ* = 1045 nm; 457 fs; 100 kHz; 113 J cm^−2^;	10–100 nm	NPs	^83^
23	Au-plate	Water	Au@graphitic carbon	Q-switched Nd:YAG	*λ* = 1064 nm; 7 ns; 10 Hz; 80 mJ pulse^−1^; focal length 30 cm	<50 nm	Spherical	^38^

The noble metals, such as Pd, Pt, Ag, and Au, derived from PLA have distinct shape, size, surface plasmon resonance (SPR), zeta potential, and enhanced optical properties, which allow them to be widely used in optics, materials science, and drug delivery.

#### Alloy and core-shell nanoparticles

Bimetallic or alloy NPs show remarkable performances compared to their monometallic counterparts and have received increasing interest in the scientific community^66^. For example, Yang et al. correlated the catalytic behavior of NiPt alloy NPs with pristine Ni and Pt-NPs for borazane hydrolysis and demonstrated the high catalytic performance of NiPt-NPs^67^. Likewise, NiPt and NiRu NPs exhibited improved electrocatalytic performance toward the dehydrogenation of ammonia compared to their individual counterparts^68^. In addition, the alloy NPs act as multifunctional materials as they comprise the combined properties of the respective individual metals. A typical example of FeAu alloy NPs involves the combined unique properties of Au-NPs, such as SPR and magnetic behavior of Fe NPs, and henceforth finds applications in the magneto-optical and catalysis fields^69,70^. Furthermore, the added advantage of the bimetallic NPs is that alloying utilizes inexpensive, earth-abundant transition metal ions, which reduce the potential use of noble metals, including Pt and Pd, leading to cost-effective materials.

Among the various alloyed NPs produced by PLAL, Ag-Au is one of the classical examples owing to the similar lattice constants of Ag and Au, which made them completely miscible in the whole concentration range^71,72^. Furthermore, both Ag and Au-NPs provide strong SPR bands in the visible region, and the SPR of Ag-Au alloy could be altered by changing the ratio of Au/Ag. A series of colloidal Ag-Au alloy NPs with various concentrations have been prepared through the ablation of Ag-Au targets (different concentrations) in water by Barcikowski et al.^73^. The formation of alloy NPs of certain elements is highly challenging via conventional synthetic routes. For example, it is impossible to form alloys such as Au–Fe and Pt-Fe under ordinary ambient conditions due to their wide miscibility gap. Conversely, the hydrothermal method and wet chemical strategies generally occur near the thermodynamic equilibrium that could provide a hybrid structure of aggregated phases instead of alloy NPs.

As compared to conventional chemical strategies, PLAL is an apt metastable process to conduct alloy formation at extreme conditions, including high-pressure and high-temperature laser plume, and generally, the reaction happens above the thermodynamic equilibrium. Au–Fe-alloy NPs constructed by the PLAL of the Au_73_Fe_27_ target in ethanol were investigated for multimodal imaging and diagnostic applications by Amendola et al.^74^. The formation mechanism of the Fe–Au alloy involves the ejection of melted drops from the target. Further evidence from UV-vis absorption spectra showed that after alloying with Fe, Au-NPs tend to provide SPR peaks with reduced intensity. In the meantime, magnetic study results revealed the strong magnetic responsive nature of the Fe–Au alloy even after introducing a minor amount of Fe in the Au lattice. From the observed data, they firmly concluded that the PLAL method can overcome the thermodynamic limitations and make it more feasible to obtain Au–Fe alloys at ambient conditions. A similar study on the production of spherical Fe–Au alloy NPs using the PLAL process has been reported by Fuse et al.^75^. In recent years, the colloidal solutions of Pt-NPs–graphene nanosheets, Pt–Ir alloy, and Pt–Au in acetone have been fabricated through femtosecond laser ablation, which can find applications in electrocatalysis, sensors, and energy storage^76–78^. Based on the concepts introduced by Amendola, various alloy NPs including Ni–Fe, Ni–Ti, Sm–Co, Al–Cu–Fe, and Au–Ge have been successfully constructed from their respective targets in liquid media^77,79,80^. For instance, the fs-laser ablated Pt–Ir alloy NPs formed without stabilizing or chemical agents show an average particle size of ~26 nm. The high stability of the Pt-Ir colloid was revealed by its zeta potential value of ~45 mV and hydrodynamic diameter of ~63 nm, respectively^77^. Interestingly, the fs-laser ablated NiTi NPs show a large particle size distribution of ~10–200 nm. Furthermore, it showed interesting effects that include the phase transitions of austenite and weak martensite in the generated NiTi NPs. Moreover, the phase transition temperature was much lowered (40 K) than the bulk target^79^.

Solvents employed in the PLAL process also play a huge role in determining the internal structural constituents of bimetallic NPs. For instance, when water or aqueous media was employed as solvents in the construction of the Au–Fe alloy, it could form Fe_3_O_4_ shell over the surface of the Au particle, whereas the introduction of organic media including methacrylate, acetonitrile, or acetone solvents produces Fe@Au core-shell structure without further oxidation of iron. Also, in such a case, no alloy NP formation occurs despite the use of the Au–Fe-target. While combining various compositions of elements in both aqueous and organic media, the core-shell morphology is more predominant, which may be attributed to the solvent–metal and solvent–metal-oxide interactions. It is anticipated that NP formation is more favored during the PLAL process owing to the kinetically controlled process at the initial stage. Then, the internal phase transformation of NPs occurs owing to metal-solvent interaction. While at high temperature and pressure, the cavitation bubbles finally collapse, which leads to NP formation in unstable thermodynamic conditions, favoring the aggregation of phases and core-shell structure. Based on this mechanism, plenty of core-shell NP structures including Zn@ZnO, Au@Al_2_O_3_, Ag@SiO_2_, Fe@FeO_*x*_, Fe@C, Au@GC, and Au@CdO have been successfully fabricated through the PLAL process^38,81–85^. The different physicochemical, morphological, and laser parameters, including the laser sources, solvents, fluences, and irradiation times of PLA-processed metals and alloy materials are provided in Table 3.

In summary, we have discussed the various plausible formations of alloy and core-shell NPs, such as Ag-Au, Au-Pt, Au-Fe, Pt-Fe, Ag@SiO_2_, and Fe@FeO_*x*_ from bulk alloy using PLA process in various solvent media. During alloy formation, the PLAL occurs either directly inside the cavitation bubble or plasma plume through the amalgamation and evolution of the NPs. The facile preparation process and controlling NPs size by changing the laser pulses are the major advantages of this versatile PLAL preparation of alloys. In addition, the absence of any unwanted ions/impurities in the final alloy and core-shell product makes this preparation more feasible for making several other metal alloy nanostructures.

#### Oxide materials

In general, the prime and most common products of the ablation process in liquid media are the metal-oxide NPs obtained through the reaction of ablated target metals with solvent media. As mentioned earlier, during the ablation process in the presence of laser-produced plasma, if the target material is a noble metal (Pt, Pd, Au, or Ag) or materials such as silicon or carbon, pure metal NPs were formed as the end product. This, in turn, means that the chemical reaction of target metals with a liquid medium is highly restricted in such cases. When surfactant or additive agents are introduced in liquid media to stabilize NP formation in such circumstances, metal-oxide or hydroxide NPs are formed as the ablated products. To avoid the aggregation effect and improve the stability of NPs, the use of co-ligand or surfactant is also beneficial in the PLAL process. For instance, Zeng et al. generated ZnO and TiO_2_ NPs using a 1064-nm Nd:YAG laser to irradiate Zn and Ti-metal targets in an aqueous solution using SDS as a surfactant^86,87^ and employed them as promising candidates for photocatalytic and photovoltaic applications. A spindle-link β-Ga_2_O_3_ NPs could be developed by the PLAL process of the Ga target in water using CTAB as a surfactant by Huang et al.^88^. Interestingly, using a similar Nd:YAG laser source, metal targets like Ni, Co, and Al in the water itself produce their respective NiO, Co_3_O_4_, Al_2_O_3_ NPs without the addition of any surfactants through the PLAL process^89–91^.

**Fig. 12:**
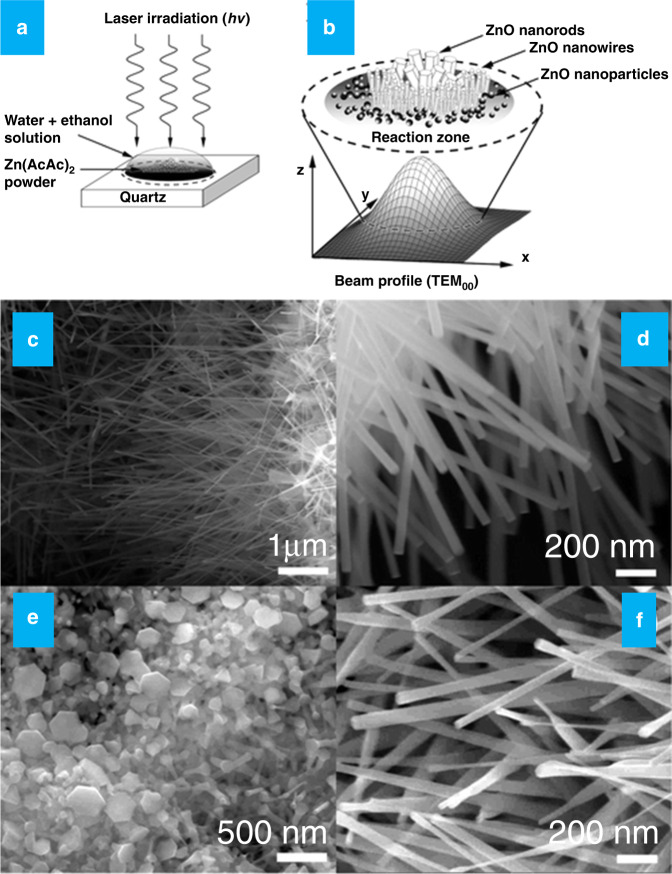
CO_2_ laser assisted synthesis of ZnO nanostructures. **a**, **b** Schematic illustration of the synthesis (laser-induced decomposition) and reaction zone of various ZnO nanostructures, **c**, **d** SEM images of ZnO nanowires after laser irradiation with a pulse energy of 20 W for 5 s, and **e**, **f** SEM images ZnO nanonails after laser irradiation with a pulse energy of 20 W for 2 s^92^. Copyright (2021) American Chemical Society

In certain cases, during the ablation process, the liquid media could also act as targets to absorb laser energy sources and generate colloidal metal-oxide NPs. As an example, Fauteux et al. developed a series of ZnO nanostructures using a new type of CO_2_ laser source for ablating the zinc acetylacetonate (Zn(AcAc)_2_, a hydrated metal complex) in water and alcohol solvents, as shown in Fig. 12a, b^92^. After irradiating with a CO_2_ laser of *λ* = 10.6 μm for a few seconds in Zn(AcAc)_2_ of a water-ethanol solution, it produces ZnO nanorods and nanowires, respectively, along with ZnO NPs near the irradiated place, as shown in Fig. 12b. The formation mechanism for ZnO involves the oriented attachment in which small particles are aggregated to form rod-shaped bigger solid particles through the self-assembly process. Interestingly, the length and width of the formed nanowires change with the irradiation time, as shown in Fig. 12c–f. Especially, the growth of ZnO nanowires was uniform during longer irradiation times and less uniform in the case of shorter irradiation times. Meanwhile, ZnO nanocrystals have been constructed by Henley and his group by means of introducing a novel approach that included the PLAL process in a hydrothermal configuration^93^. They employed a KrF excimer laser source with a λ of 248 nm to irradiate precursor solutions, zinc nitrate hexahydrate, and hexamethylenetetramine, in DI water to generate the nanocrystals. Furthermore, they observed the growth process of NPs in the presence of laser-induced plasma controlled by Ostwald ripening, which led to bi, tri, or tetrapod-like nanostructures. Based on these concepts, different metal oxides (MOs) and hydroxides NPs, such as CuO, FeOOH, CoO, MgO, and FeO, have been developed^94–96^. A series of MOs and sulfides have been prepared via laser ablation of metal targets in the suitably selected media^96^. The corresponding MOs, such as CuO, CoO, FeO, ZnO, and MgO with a hollow nanospherical morphology are readily formed in water/ethanol medium through the Kirkendall mechanism. The metal sulfides, including PbS and ZnS, formed by ablating the metal targets (Zn and Pb) in mercaptoethanol provide hollow NPs that confirms that the PLAL is a versatile technique to achieve various nanostructures.

The PLA process of titania (TiO_2_) NPs is crucial since it is one of the prime candidates for photovoltaic and photocatalytic applications. Seto et al. synthesized TiO_2_ NPs by irradiating the Ti-metal target using a nanosecond PLA process in an inert He atmosphere^97^. Tsuji et al. introduced the laser oven process to prepare TiO_2_ NPs by means of ablating the Ti target in a tubular furnace (O_2_ atmosphere)^98^. Furthermore, they controlled the shape and size of the TiO_2_ NPs by varying the temperature of the tubular furnace, which provided an average particle diameter in the range from 11 to 24 nm at 800–1200 °C. Using Nd:YAG at 1034 nm over the Ti target in water produced TiO_2_ NPs, which showed excellent photocatalytic and antibacterial activities^99^. The improved photocatalytic behavior of Ag/TiO_2_ NPs has been observed by Zhou et al. by means of ablating the Ag and Ti plates in ethanol–water mixture through an Nd:YAG laser (1034 nm)^100^. Recently, the conversion of anatase TiO_2_ to rutile TiO_2_ NPs by varying the surfactant concentration and ablation time in a PLAL process has been reported by Chaturvedi et al.^101^.

Magnetic NPs comprised of iron-related compounds, especially iron oxide, have received huge consideration for their extensive applications in biomedical, material science, biotechnology, environmental, and engineering fields because of their excellent catalytic, biocompatible, and magnetic behaviors^102^. Henceforth, the extraction and synthesis techniques of iron-related compounds have become popular in the late 90s. The synthesis of Fe NPs through PLAL is free from toxic starting materials, and they possess outstanding chemical reactivity. Among the different synthesis methods employed, PLAL is one of the finest processes to obtain high-purity iron oxide-based NPs. The preparation of Fe NPs through PLAL has been extensively investigated using different laser fluences, solvents, and irradiation time. Due to the high reactivity of Fe vapors, which react with surrounding liquids during the PLAL process, it can afford various carbonization and oxidation states in a nanosecond. Henceforth, choosing the right liquid medium and laser energy source is highly essential for the formation of Fe NPs via the PLAL process. Amendola et al. derived multifunctional iron oxide NPs from the Fe metal target in a water medium through PLAL^103^. The magnetic Fe_3_O_4_ and Fe_3_C NPs have been derived from the PLA of the Fe foil in ethanol by Franzel et al.^104^. The prepared Fe NPs in water and ethanol have possessed the maximum super magnetization values of 80 and 124 emu.g^−1^, respectively. In this process, the NP formation mechanism comprises small embryonic particle growth followed by their coalescence into macro particles. Following Franzel et al., several research groups have investigated the effects of solvents on the formation of FeO_*x*_ nanostructures by ablating the bulk targets such as Fe_3_O_4_, Fe_2_O_3_, and Fe in water which produces their corresponding iron-oxide (FeO, Fe_3_O_4_) NPs. Two small particle diameters were achieved through this process including 1 and 5 nm using Nd:YAG laser. The small diameter particles were evenly distributed, whereas large diameter (5 nm) particles were agglomerated, which could disperse uniformly using the protective agent poly(*N*-vinyl-2-pyrrolidone) (PVP). The dispersity of large particles depends on the PVP concentration. A similar observation has been found for fabricating FeO NPs from the pure Fe target in PVP solution. The particle size of FeO can be controlled by PVP concentration. The optical absorption analysis confirmed the redshift for FeO NPs with improved optical features. They concluded that PLAL is an appropriate technique for the fabrication of dome NPs with special features that conventional techniques could not easily obtain^105,106^. Recently, Svetlichnyi et al., fabricated a series of iron oxide nanostructures including Fe_3_O_4_, Fe_2_O_3_, and FeO_*x*_ via PLA using an Fe-target in water^102^. Kanitz et al. investigated the ultrashort PLA of the Fe-target in different solvents, including acetone, water, methanol, ethanol, and toluene, and studied the relationship between the liquid properties and the ablation rate on the formation of Fe NPs^107^. The NPs generated by the ultrashort PLA possessed better polycrystallinity than the short PLA-produced NPs. The significant parameters for the PLAL process that derived FeO_*x*_ and other metal-oxide nanostructures are tabulated in Table 4.

**Table 4:** Various laser parameters, including the laser source, fluence, irradiation time, and solvent of PLA-processed metal-oxide and nonoxide materials

No.	Metal targets	Solvent	Products	Laser source	Condition	Particle size (nm)	Morphology	Ref.
1	Zn-plate	Aqueous solution of SDS	Zn/ZnO	Nd:YAG	*λ* = 1064 nm; 10 ns; 10 Hz; 70 mJ pulse^−1^; focal length 150 mm	18.1–44.5 nm	Spherical	^86^
2	Ti-metal plate	Aqueous solution of SDS	TiO_2_ nanocrystals	Nd:YAG	*λ* = 355 nm; 5 ns; 10 Hz; 150 mJ pulse^−1^; focal length 150 mm;	3 nm	Spherical	^87^
3	Ga-metal plate	Aqueous solution of CTAB	GaO_2_	Nd:YAG	*λ* = 1064 nm; 5 ns; 10 Hz; 100 mJ pulse^−1^; irradiation time 20 min	90–220 nm	Spindle NPs	^88^
4	Co, CoO, and Co_3_O_4_ powder	Water	Co_3_O_4_	Focal lens—Nd:YAG	*λ* = 355 nm; 60 min; 10 Hz; 30 mJ pulse^−1^; focal length 100 mm;	20–100 nm	Colloidal NPs	^89^
5	Al-rod	Water	Al_2_O_3_	Nd:YAG	*λ* = 532 nm; 2 s ns; 12 Hz;	–	NPs	^90^
6	Ni-metal	Aqueous solution of H_2_O_2_	NiO	Nd:YAG	*λ* = 355 nm; 30 min; 10 Hz; focal length 250 mm;	8 nm	NPs	^91^
7	Ti-metal plate	Water	TiO_2_	Q-switched Nd:YAG	*λ* = 1064 nm; 10 ns; 10 Hz; 5 J cm^−2^; focal length 20 cm	20–60 nm	Spherical	^99^
8	Fe-target	Water	Fe_3_O_4_, Fe_2_O_3_, and FeN_x_	Nd:YAG laser	*λ* = 1064 nm; 7 ns; 20 Hz; 150 mJ; focal length 50 cm	80 nm	Agglomerated spherical nanoparticles	^102^
9	Cu-foil	Water	CuO	Frequency doubled Nd:YAG laser	*λ* = 532 nm; 6 ns; 10 Hz; 2 J cm^−2^; focal length 35 cm	–	–	^108^
10	Cu-Plate	Water	Cu@Cu_2_O	Nanosecond-pulsed Nd:YAG laser	*λ* = 1064 nm; 30 min; 10 Hz; 35 mJ pulse^−1^;	2–7 nm	Colloidal nanoparticles	^109^
11	Cu-Plate	DMSO	CuS	Q-switched Nd:YAG	*λ* = 1064 nm; 7 ns; 10 Hz; 0.5–5 J cm^−2^; focal length 100 mm	45–825 nm	Spherical nanoparticles	^35^
12	Sb_2_S_3_ pellet	Water and isopropyl alcohol	Sb_2_S_3_	Nd:YAG	*λ* = 532, 1064 nm; 10 ns; 10 Hz; 8.5–80.3 J cm^−2^;	10–30 nm	Interconnected chains of nanoparticles	^115^
13	Disk CuSbS_2_ sputter target	Acetone, methanol, ethanol, DMF, and isopropyl alcohol	CuSbS_2_	Nd:YAG laser	*λ* = 532, 1064 nm; 30 ns; 10 Hz; 0.63 J cm^−2^;	2–6 nm; 5–15 nm	Crystal structure	^116^
14	SnS-target	Water, acetone, 2-propanol, and ethanol	SnS	Nd:YAG laser	*λ* = 532 nm; 10 ns; 10 Hz; 230–300 mJ pulse^−1^; 6–24.8 J cm^−2^; focal length 20 cm;	6.7–18 nm	Spherical nanoparticles	^118^
15	MoS_2_-target	Water, n-decane	MoS_2_	Second-harmonic Nd:YAG lase	*λ* = 532 nm; 5 ns; 10 Hz; 230–300 mJ pulse^−1^; 1–10 J cm^−2^;	–	Lamellar structure	^123^
16	2H-MoS_2_ and WS_2_ targets	Water	MoS_2_ WS_2_ quantum dots	Femtosecond Nd:YAG laser	*λ* = 800 nm; 50–100 ns; 1 kHz; 0.77 J cm^−2^; focal length 3 mm;	1–5 nm; 2.6 nm	Quantum dots	^124^
17	PbTe-powder	Water	PbTe NPs	Nd:YAG	*λ* = 266, 532, and 1034 nm; 50–7 ns; 10 Hz; 7–28 J cm^−2^; focal length 400 mm;	4–10 nm	NPs	^129^

Similar to Fe, Cu has gained huge attention in the fields of metallurgy, nanoelectronics, optoelectronics, and catalysis owing to its abundance and electrical, mechanical, optical, and catalytic properties. In the late 90s, Jordan et al. studied the laser ablation of the Cu target in water using an Nd:YAG laser operating at a pulse length of 6 ns and wavelength of 532 nm to generate Cu-NPs^108^. The aging effect of Cu@Cu_2_O NPs fabricated by the PLA of the Cu-plate in water was systematically investigated by Swarnkar et al.^109^. The formed cactus-like Cu@Cu_2_O NPs had a 7-nm diameter with a bandgap energy of 2.24 eV. The same group developed Cu@Cu_2_O nanowires through PLA in water, which showed improved antibacterial activity^110^. Few other reports showed the unique morphology of Cu-NPs through the PLA of the Cu target in aqueous solutions^111–113^, which are discussed in detail as follows: The PLA of Cu/Cu_2_O NPs in ethanol was prepared and studied for the effect of low quantity of acid-base and oxidizing species on the structure, composition, and morphology of the formed NPs. While using water as a solvent in PLA, owing to NaOH and H_2_O_2_ species, a cubic Cu_2_O was formed rather than Cu NPs. The morphologies of the Cu_2_O could be changed with respect to NaOH and H_2_O_2_ in water, such as leaf-like CuO and sheet-like CuO formed during the PLA process on Cu target. These observations undoubtedly confirm that PLA is a major technique to generate different shapes of Cu NPs. However, the ablation efficiency analysis was performed under different numbers of pulses per burst in the range between 1 and 40. It was observed that the ablation efficiency (4.84 µm^3^ µJ^−1^) increased by 20% for 3 pulse/burst compared to the nonburst regime. This study provides the advantages of the burst regime for forming PLA-based Cu NPs. Fascinatingly, cube- and nanorod-shaped copper cyanide and Cu_2_O@graphitic carbon NPs have been developed by the PLA of the Cu-plate in acetonitrile and water by Begildayeva et al.^10^. Especially, the PLAL-produced Cu_2_O@graphitic carbon layer showed a superior catalytic reduction of nitrophenol and nitrobenzene.

Based on the discussions mentioned earlier, it is concluded that the nature and properties of stable colloidal MO NPs, such as ZnO, TiO_2_, Fe_3_O_4_, Cu_2_O, and ZrO_2_ produced via PLAL depend on the laser fluence, energy, and the number of laser pulses in liquid. The prepared colloidal oxide NPs comprise high absorption and relatively low bandgap values, confirming their better optical properties. For large-scale commercial production, the reusability and stability of these MO NPs should be checked under various laser conditions and solvent media.

#### Nonoxide materials

Nanometer-sized metal sulfides can be applied to a wide range of applications in several fields, including photocatalysis, antimicrobial activity, energy conversion/storage devices, and optoelectronics, due to their specific physicochemical behaviors. Between various synthesis approaches, the PLAL of metal sulfides is of great interest due to their high quality, simplicity, and product purity. In the beginning, Niu et al. prepared various metal sulfide NPs, such as ZnS, PbS, and CuS, through the ablation process of metal targets in sulfur precursor liquid media, including β-mercaptoethanol, 1-dodecanethiol, and dodecyl mercaptane^96,114^. The effect of laser fluence on the formation of CuS NPs has been studied by Khademian et al.^35^. For this process, they used an Nd:YAG laser with 1064 nm at 7 ns pulse to irradiate the Cu target in DMSO, and the prepared CuS exhibited a bandgap between 3.77 and 3.94 eV. Very few reports on the generation of antimony sulfide (SbS), copper-antimony sulfide (CuSbS_2_), arsenic sulfides (As_2_S_2_ and As_2_S_3_), and SnS NPs through the PLAL process have recently been reported^115–118^. More recently, our group has fabricated ZnS nanospheres using the PLA of the Zn target in DMSO without using any additional sulfur source and reducing agents. DMSO was employed both as a solvent as well as a sulfur source for the production of ZnS^34^.

Meanwhile, the PLAL process of CdS, a semiconducting material with various nanostructures, including quantum dots, thin films, and nanowires, has been vastly studied. Vitukhnocsky et al. successfully prepared quantum dots of CdS and ZnSe in liquid media (diethylene glycol and ethanol) and achieved average particle sizes between 10 and 20 nm^119^. Likewise, CdS NPs with outstanding colloidal stability even at high laser fluences have been developed by Gong et al.^120^. The effect of various laser parameters such as pulse width, wavelength, fluence, and surfactant in liquid, on the formation of CdS NPs has been investigated by Darwish et al.^121^. Recently, a series of colloidal NPs of CdS, SnS, and CuS have been prepared efficiently by ablating their respective metal targets in sulfur precursors containing DMSO, and their antimicrobial activity against various microorganisms has been studied^36^.

Owing to their excellent chemical and physical properties, 2D graphene-like MoS_2_ metal chalcogenides are considered candidate materials for numerous applications. The bonds S–Mo–S appear as a sandwich structure held together by weak van der Waals forces, which can be broken easily. Furthermore, it comprises a sizable intrinsic bandgap for various energy applications. Similar to CdS, MoS_2_ has also been prepared in different nanoforms through the PLAL process. As an example, the thin films of MoS_2_ over an alumina substrate have been prepared through the KrF excimer laser ablation process in the early 90s^122^. A colloidal MoS_2_ fullerene-like NPs with an average diameter of 10–15 nm has been prepared through the ablation process of the MoS_2_ target in water by Compagnini et al.^123^. Also, Li et al. constructed MoS_2_ quantum dots through the femtosecond PLA process of MoS_2_ targets in water^124^. Due to the excellent purity of the prepared single-layered MoS_2_ quantum dots, it finds plausible electrocatalytic application toward hydrogen evolution reaction. The different physicochemical, morphological, and laser parameters, including laser source, solvents, fluences, and irradiation time of PLA-processed oxides and nonoxide materials are provided in Table 4.

The preparation of metal selenides through the PLAL process is highly complicated due to the high reactivity of selenium and tellurium precursors in liquid media, readily forming their respective oxides. Henceforth, reports on the PLAL process of metal selenide formation have been limited. The type II–IV semiconducting materials such as CdSe, ZnTe, and CdTe NPs with an average radius of 5–6 nm have been produced through the PLAL process in various liquid media by Semaltianos et al.^125^. Likewise, the spherical NPs of CdSe and CdTe with an average radius of 3–7 nm have been reported by Ruiz et al.^126^. CdSe quantum dots have been prepared through PLA in superfluid helium by Minowa et al.; they reported the improved luminescence property of the CdSe quantum dots^127^. The effects of laser parameters on the formation of Ag_2_Se, PbTe, and AgInSe_2_ in PLA have also been investigated and reported elsewhere^128–130^. For instance, an innovative laser-based solid-liquid ablation method was used for fabricating silver selenide (Ag_2_Se) NPs from Se powder and AgNO_3_ using ethylenediamine and 2-propanol. Homogeneous spherical particles with an average size of 30 nm were formed via this process^128^. Similarly, well-crystallized PbTe NPs with an average size of 4–6 nm were produced through the PL fragmentation of PbTe bulk powder^129^. The effects of various experimental parameters, such as treatment duration, wavelength, and output energy have been studied. Though uniformly grown homogeneous particles with specific shapes are formed, their production efficiency is very low.

Here, we summarize the fabrication, laser parameters, and properties of various sulfide, selenide, and telluride NPs by PLAL and discuss their feasibility in various electro and photocatalytic processes. Effects of various laser times (fs and ns), liquid media, and laser wavelengths (532 and 1064 nm) on the shape, optical, and structural properties of the metal halide NPs are summarized in more detail. A few works on the production of metal sulfides and carbon composites without any additional external source for sulfur and carbon because the solvents used in the PLAL act as sulfur (e.g., DMSO) and carbon sources (e.g., carbon-rich solvents like hexane) are discussed. This should be extended to fabricate a wide range of metal sulfides and other nonoxide materials such as carbides and nitrides.

There are some minor disadvantages to the fabrication of metallic nanostructures using PLA. For example, long exposure to laser ablation leads to large quantities of NPs in the colloidal suspension, leading to blocking the laser energy and laser path, which is adsorbed by generated NPs instead of the surface of the metal target. This leads to hinder the rate of PLA process.

### PLIL process

Usually, the bottom-up and top-down approaches involve the interactions between electrons, photons, and ions with materials for the construction of metal/metal-oxide nanostructures. Owing to these interactions, there is an energy transfer between the molecules and irradiated material atoms. This section of the review article focuses on using laser–material interactions to control the structure at the nanometer scale. Recently, fast and ultrafast pulsed lasers (ns, ps, and fs) have received considerable attention to draw a new perspective for fabricating NPs. While irradiating with the pulsed lasers, plasmonic NPs could be rapidly heated to high temperatures. Henceforth, the thermal effects as functions of laser wavelength, pulse width, and pulse energy are responsible for melting, fragmentation, and reshaping processes. Various nanostructures, including metal NPs, MOs, metal nanocomposites, carbon, and metal halides, have been assessed for their stability to the PLIL process; however, identifying the particle size and distribution of the resulting nanostructures remains challenging because identifying the structure–stability relationship requires extreme experimentation^131^. Moreover, the structure–stability phenomenon is a critical tool for understanding the purity, particle compatibility, and firm role in photo- and electrocatalytic applications. Moreover, the structure–stability relationship in the PLIL process is further advantageous for fabricating innovative metal and metal composite nanostructures.

As described earlier in the PLAL process, the laser source is focused on the solid metal target immersed in a solvent, which leads to the evaporation of the materials that are converted into an intensifying plasma plume surrounded by the solvent. On the other hand, PLIL is firmly related to the irradiation of colloidal suspended particles in a solvent medium in which the laser energy is cautiously absorbed by the NPs, which leads to their heating without transferring their thermal energy to the solvent medium. The phase changes and temperatures acquired by the formed NPs strongly depend on the laser fluence, refraction index, optical, and thermodynamic properties of the solvent. During the reaction, the heat energy of NPs reduces to room temperature and recrystallize. Apart from NP reshaping, this process is also used to form alloy and composite NPs by irradiating mixed colloidal suspensions.

#### Metallic nanoparticles

Recently, metallic nanostructures fabricated by PLI have received great interest owing to their widespread applications in the fields of electronic, sensing, catalytic, and optophotonic devices. By means of nanosecond, picosecond, or femtosecond laser irradiation, the shape can be varied, and the size of the desired metal particles can be reduced either in an aqueous medium or supported substrates. In particular, the size reduction of the NPs can be explained by melting/heating and the excitation of robust surface plasmon transition through the laser energy. To date, numerous reports were available on the feasibility of particle size modification through PLI.

**Fig. 13:**
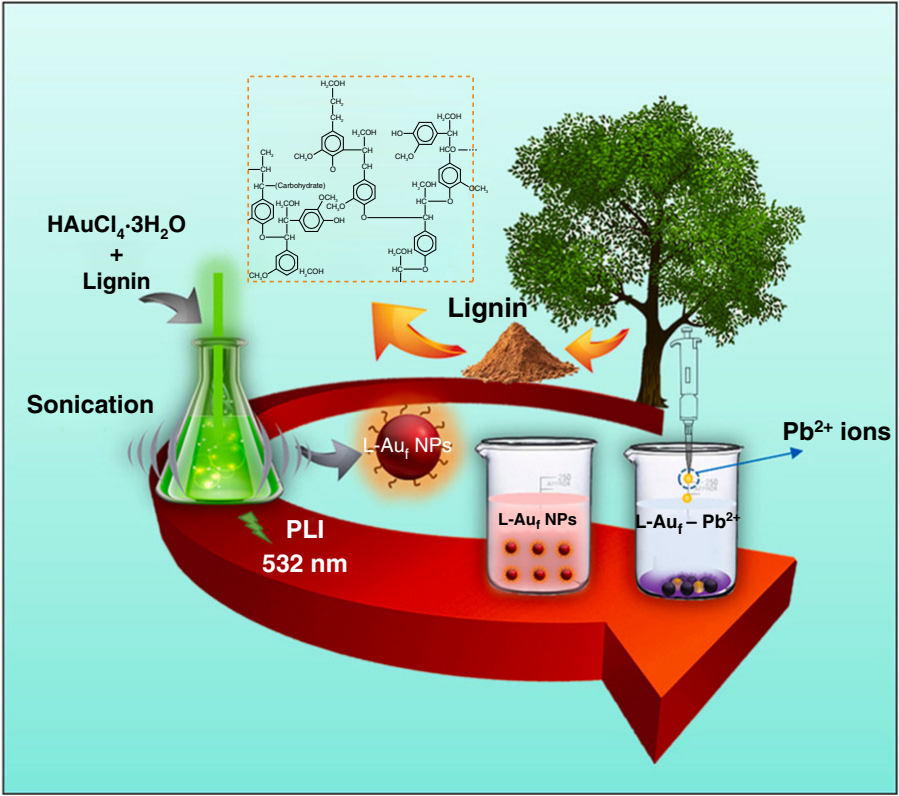
Schematic design for preparing lignin-mediated Au-NPs using PLI for calorimetric Pd^2+^ detection^14^. Copyright (2021) Elsevier

The fabrication of metal NPs mainly depends on the types of laser energy source, solvent, and irradiation time employed. In the later 90s, Kurita et al. formulated the size reduction of Au-NPs with <20 nm diameter through the second-harmonic PLI process using an Nd:YAG laser (532 nm) source in water^132^. The prepared Au-NPs are spherical and formed by heating the colloidal suspension within a short time of laser irradiation. The formation mechanism of Au-NPs by the Nd:YAG laser is realized by the initial surface plasmon absorption of Au-NPs followed by poor energy loss of the heated Au-NPs in water. The same group proposed the formation of Ag-NPs through PLI in which the formation mechanism remained unclear^133^. Interestingly, Prevo et al. proposed a way to form Au nanoshells with the desired size through PLI in aqueous media^134^. In this process, a femtosecond laser pulse was applied to a colloidal sol, which allowed the smallest nanoshells to reach the highest temperature and produce spherical NPs quickly. The ultrashort-pulse irradiation of Au-NPs in polyvinyl alcohol solution has produced Au nanorods that possessed excellent chromaticity^135^. Very recently, the PLI production of functionalized Au-NPs with the aid of lignin matrix in water has been proposed by Yu et al.^14^. In this study, a nanosecond (10 ns) pulsed 532 nm laser was utilized to irradiate various concentrations of HAuCl_4_ and lignin solution to obtain the desired lignin-functionalized Au-NPs (Fig. 13). The desired functionalized Au-NPs were subjected to the calorimetric sensing of various heavy metal ions, including Pb^2+^, Cu^2+^, Fe^3+^, Co^2+^, Ba^2+^, Cd^2+^, and Hg^2+^, and they effectively detected Pb^2+^ from various ions.

Similarly, the PLI process of other metal NPs such as Cu and Co has also been reported rarely. A nanosecond-pulsed Nd:YAG laser irradiation of Cu-NPs in ethanol solution created the Cu submicrospheres using a fixed pulse rate and energy fluence of 5 Hz and 300 mJ cm^−2^ pulse^−1^, respectively^136^. Furthermore, it possessed wide solubility in different commercial lubricants, which might be attributed to its improved tribology properties, such as excellent water resistance and reduced friction properties. On the other hand, the synthesis of 4-nm Co-NPs has been reported by Robinson et al. through the PLI of cobalt carbonyl in organic solution^137^. The size of the Co-NPs could be controlled by changing the reaction conditions, such as laser wavelength (355 and 266 nm) and ligand concentration. Using a KrF excimer laser, Lu et al. investigated the surface cleaning of various metals, including Cu, Al, and stainless steel, through PLI in the air^138^. They have observed that the short-pulse duration (20 ns) and wavelength (248 nm) are essential for laser surface cleaning, and it effectively cleaned the Cu metal plate compared to other metal substrates.

Recently, the PLI process has been widely used to fabricate various metal alloys due to their synergistic behaviors, tunable properties, and promising applications of metal alloys over metal NPs. Among the different alloys, Au and Ag combinations are gaining a great deal of consideration owing to their tunable plasmonic properties, which find applications in photo-imaging, drug delivery, and photothermal therapy. For instance, the Au-Ag nanocages were prepared through the PLI-aided galvanic replacement of Ag nanocubes in HAuCl_4_ solution, which showed improved photothermal stability^131^. The formation of Ag-Au nanocages was confirmed by means of the localized SPR peak centered at 750 nm. Very recently, Au-coated Ag nanoplates have been constructed through PLI using 6-ns FWHM laser pulses and second-harmonic 532-nm Nd:YAG irradiation^139^. Furthermore, the laser energy and irradiation time were fixed at 25 mJ pulse^−1^ and 1 min, respectively. The morphology can be varied from triangular (22:1 for Ag:Au) to spherical based on the ratio of the Ag ion released during PLI (4.5:1 for Ag:Au). The prepared Au-coated Ag nanoplates showed controllable antibacterial activity through PLI.

A few reports concerning the alloys of Au with d-block transition magnetic elements (Co, Fe, and Ni) have been reported through PLI. When these d-block transition elements are alloyed with Au, under equilibrium conditions, they are immiscible; however, their nonequilibrium phases are of great consideration owing to their promising diverse optical, magnetic, and catalytic properties. For instance, the single-phase bimetallic Au–Co alloy has been synthesized through the PLI process using an Nd:YAG laser in ethanol with a pulse fluence and rate of 100 mJ cm^−2^ pulse^−1^ and 30 Hz, respectively^140^. The sub-micrometer particle with an average particle size of 230 nm showed ultimate magnetic properties at a moderate temperature (Coercivity of 102 Oe at 300 K). Likewise, the Au-Si composite was synthesized by the nanosecond PLI process using Si (100) surface^141^. The formation mechanism involves both top-down and bottom-up effects, which include the decomposition of colloidal suspension into smaller particles followed by the self-assembly of small particles into larger particles. The Cu-Pd alloy has been synthesized using a second-harmonic pulsed Nd:YAG laser in ethanol operated at laser parameters of 10 Hz, 5 ns, and 532 nm, respectively^142^. The reason behind the formation of the Cu-Pd alloy might be attributed to the fact that the alloyed Cu and Pd-NPs are expected to show exceptional cooperation for the hydrogenation reaction in which Cu acts as a catalyst. Very recently, spherical Ni-Pd alloy has prepared by Yu et al. using a new hybrid technique of combined PLI and ultrasonication processes^1^. The prepared PLI-based Ni-Pd alloy has been characterized for its application in HER, and it showed excellent performance in KOH solution with an overpotential value of 38 mV at 1 mA cm^−2^. Also, the PLI-processed multiphase core-shell liquid has been used for Ga-In alloy quantum dot preparation, which showed an outstanding power conversion efficiency of 17.18% for perovskite solar cells^143^.

As stated above, this laser-assisted process is used for generating various nanosized materials, such as Au, Cu, Co, Ag-Au, Au-Co, Cu-Pd, and Ga-In NPs, at nano, micro, and submicron levels via laser irradiation. The morphologies and size of these NPs showed a significant dependence on the experimental and laser parameters. For any specific application like catalytic, drug delivery, and biotechnology, the carrier metallic NPs obtained using PLIL should be in optimal size and comparable with biomolecule size. Henceforth, the PLIL approach for the fabrication of metal and alloy NPs <5 nm is of technological and scientific interest in using them for catalytic applications.

#### Metal-oxide and their composite materials

Oxide/oxide composite-based semiconductor material production through PLI has received huge attention in recent years owing to its tunable physicochemical properties, improved functional properties, and structural stability. Also, they exhibited excellent in-built magnetic, optical, and electrical properties, which made them useful in photocatalysis, electrocatalysis, and energy storage and conversion devices. The MOs and their composites obtained by PLI are very limited. Wang et al. developed spherical NPs of various MOs, including CuO, Fe_2_O_3_/FeO, Co_3_O_4_, NiO, WO_3_, and ZnO through PLI at *λ* = 355 and 532 nm by an Nd:YAG laser^140,144^. The growth mechanism of colloidal spherical CuO-NPs obtained through nanosecond laser irradiation has been experimentally and theoretically investigated by Pyatenko et al.^145,146^. Heating, melting, and fusion seemed to be responsible for the formation of the resultant spherical metal-oxide particles. Furthermore, they inferred that this technique is highly suitable to make wide ranges of spherical semiconducting MOs at the sub-micrometer scale. Moreover, PLI-processed TiO_2_ is of high interest due to the use of toxic precursors, simplicity, and the absence of expensive vacuum systems. The conversion of white TiO_2_ powder into black TiO_2_ powder has been successfully prepared by nanosecond laser pulses (10 ns) using an Nd:YAG laser in liquid media^147^. The laser wavelength and pulse rate were maintained at 532 nm and 10 Hz, respectively. It is evident from the UV-vis analysis that the irradiated black rutile TiO_2_ possessed a narrow bandgap (1.98 eV) compared to that of the white TiO_2_ (2.98 eV). When TiO_2_ is subjected to nanosecond (10 ns) laser irradiation in the air medium, it splits into a thin rutile TiO_2_ cap, a middle TiO layer, and inhomogeneous titanium oxynitride layers^148^. By means of varying the laser power (5.6, 6.6, and 7.6 W), the thickness of the TiO layer could be changed from 10 to 120 nm. Furthermore, at high laser fluences, oxygen-rich phases were predominant, whereas, at low laser fluences, the rutile TiO_2_ phase was favored. Other reports on PLI-processed La-doped TiO_2_ and graphene oxide (GO)/TiO_2_ composites have also been studied for their improved optical and catalytic properties^149,150^. Interestingly, the nonlinear properties of La-doped TiO_2_ nanorod arrays are in the order of 10^−5^ esu compared to the undoped one.

ZnO is one of the well-known semiconducting materials with an excellent bandgap, which finds diverse applications, especially in the areas of optoelectronics and catalysis. Single-crystalline spherical ZnO nanostructures were fabricated by the PLI of bulk ZnO NPs in water^151^. Based on the time-dependent laser irradiations, it was confirmed that the fusion mechanism is the ultimate reason for the formation of single-crystalline NPs. The prepared ZnO single-crystalline particles possess excellent electrical and optical behaviors and are utilized for sensitive and selective ultraviolet photodetection. Followed by the use of a short KrF excimer laser pulse (248 nm, 25 ns), hydrothermally prepared vertically aligned ZnO nanowires were converted into spherical ZnO NPs by Wang et al.^152^. A similar study has been performed by Kanakkillam et al. for the synthesis of defect-rich black-ZnO spherical NPs via nanosecond PLI in a mixed solution of ZnCl_2_ in hexamethylenetetramine^153^. The defects in the prepared ZnO were further confirmed by XPS and Raman analyses, improved catalytic activity against organic dye was demonstrated in the visible region. The same research group investigated the facile PLI synthesis of ZnO/Co_2_O_3_ in a liquid medium for the enhancement of photocatalytic activity against methylene blue (MB) dye in water^154^. On the other hand, the single-step PLI was used to produce spherical-like ZnO/PMMA nanostructures with an average particle size of 9 nm^155^.

A few studies concerning the synthesis of Au-based composites have also been investigated in the early 2000s. For instance, the core-shell silica Au-NPs composite has been constructed through near IR femtosecond laser irradiation, which acts as a strong metalloid-dielectric candidate^156^. The UV-vis spectral analysis showed two strong excitations peaks at 730 and 530 nm, which confirmed the inside-out distribution of the resultant composite structure. The fusion of Au followed by the selective melting of ZnO colloidal solution at *λ* = 1064 nm by Nd:YAG formed Au/ZnO nanonetworks by Bajal et al.^157^. According to their study, the UV-vis absorption and surface morphology of the synthesized samples are highly dependent on the laser irradiation time. Furthermore, the redshift in the Au-plasma resonance peak and large blueshift in the ZnO excitonic peak claimed the interfacial charge transfer process in the composite structure. Later, Kanakkillam et al. developed nanocomposites comprising Ag/Au-ZnO through a combined pulsed laser process, which showed an improved dye degradation activity toward MB in water^158^. A similar hybrid fabrication process involving both laser ablation and laser irradiation (PLA/PLI) was also used to derive metal composite nanostructures. A classic example of Au-CuO nanocomposite with different volumetric ratios of Au and CuO has been shown by Aazadfar et al.^159^. Interestingly, during the laser irradiation (irradiation parameters~ Nd:YAG, 532 nm, 5000 pulses, 10 Hz, 1 J cm^−2^), the Au-NPs were subjected to melting by laser heating followed by the combination of melted Au particles with nearby CuO particles to form the resultant composite structure. Recently, a series of Au/M_*x*_O_*y*_ (M = Fe, Ni, and Co) particles were also synthesized by co-precipitation followed by laser irradiation using an Nd:YAG laser at 532 nm^160^. The prepared composite samples have exhibited various morphologies and phases based on the laser width, irradiation time, and wavelength. Very few reports concerning the PLI synthesis of NiO-Ni, PdO-Pd, and SnO_2_-PMMA composites with excellent magnetic and catalytic behaviors have been demonstrated^161–163^. In addition, few reports that explore the tribology behavior of TiO_2_, Fe_2_O_3_, and ZnO spherical particles by PLIL^136^ discuss that MOs can easily destruct the friction pair’s surface under heavy load owing to high hardness.

Given the above observations, PLIL can be the optimal method for developing high purity spherical MO nanostructures with submicron size without any external reagents, like surfactants and stabilizers. Owing to these beneficial characteristics, the PLIL-derived MOs are widely used in various electrochemical and catalytic processes.

#### Metal halides, sulfides, and selenides

Transition metal halide nanostructures can be applied to photocatalytic, thermoelectric, and photoelectric applications due to their distinct magnetic, optical, thermal, and electrical properties. In this regard, considerable attention has been given to the fundamental aspects of their fabrication process, mechanisms, and advanced applications. Significant developments have been made on the rational design and fabrication of various transition metal halides with controlled composition, morphology, and structural complexity. Owing to the simplicity, ecofriendliness, and cost-effectiveness of PLI, it is widely used to construct metal halide nanostructures. Earlier, the pulsed fragmentation of copper iodide (CuI) in ethyl acetate and water was prepared by Schaumberg et al. using a second-harmonic 532-nm ultrashort laser pulse with the pulse energy and duration of 45 mJ pulse^−1^ and 6 ns, respectively^164^. In addition, the laser irradiation increased the number of CuI particles in the resultant solution with a mean diameter of 18 nm in ethyl acetate solution and 31 nm in water.

**Fig. 14:**
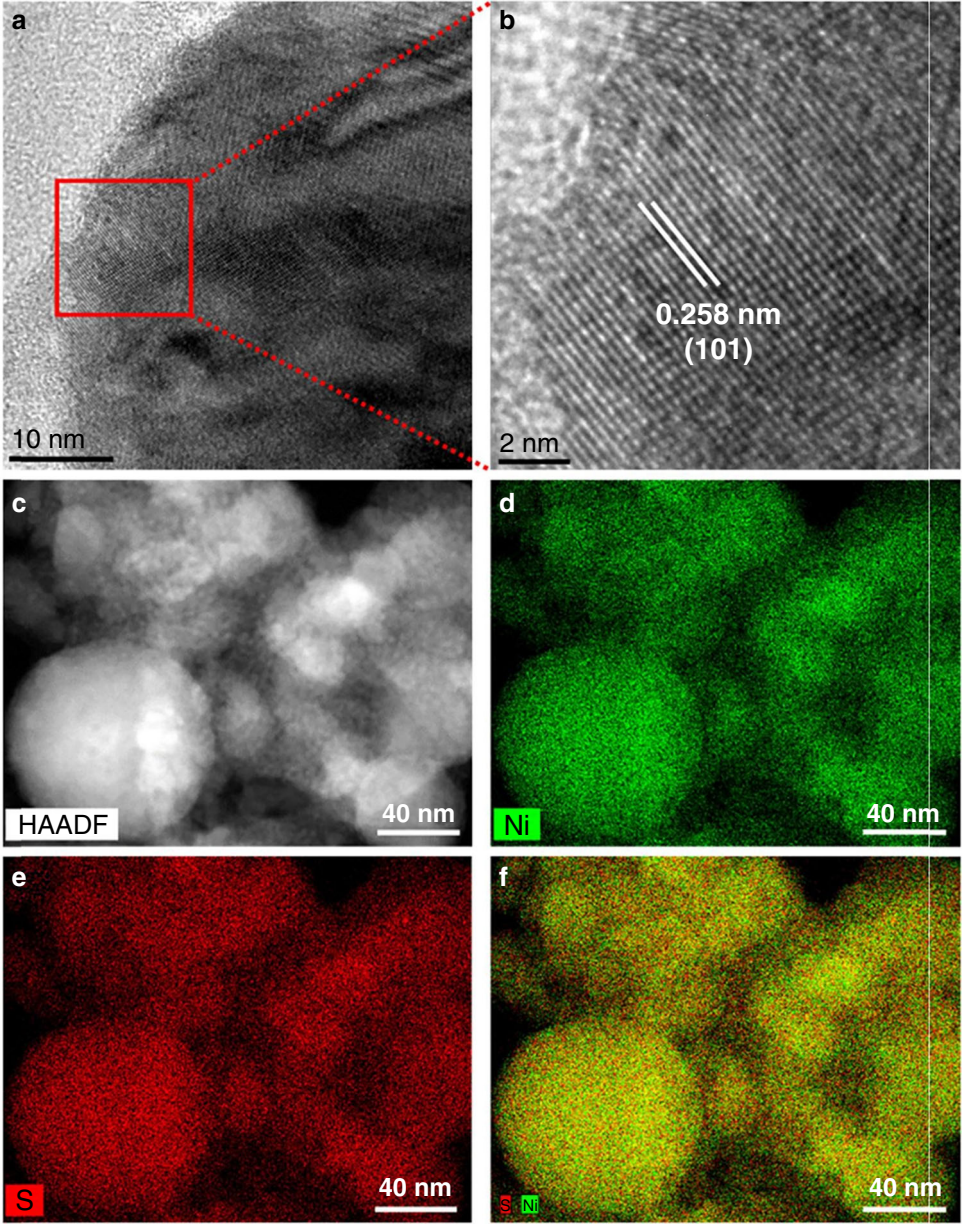
Surface structures of NiS by PLI process. **a**, **b** HR-TEM lattice fringes of NiS-4 h, **c** their corresponding HAADF image, **d**, **e** elemental mapping of Ni, S through STEM analysis, and **f** overall distribution of Ni and S elements^165^. Copyright (2021) Elsevier

Nickel sulfide (NiS) is one of the vastly studied transition metal sulfides in the areas of electrocatalysts, energy storage, and conversion devices. Hung et al. developed NiS nanostructures by irradiating the starting precursors in water with an Nd:YAG laser (1064 nm) under the pulse energy and width of 700 mJ and 4–6 ns, respectively^165^, and studied the effect of the irradiation time duration (2, 4, and 6 h). As evident from the TEM analysis (Fig. 14), the prepared NiS-4h possessed aggregated spherical morphology with an average particle diameter in the range between 40 and 60 nm, and the corresponding TEM lattice fringes pattern confirmed the (101) reflection of α-NiS (Fig. 14a, b). The spatial uniform distribution of different elements in the α-NiS structure was confirmed by HAADF-STEM analysis, as shown in Fig. 14c–f. The irradiated NiS-4h exhibited great crystallinity with high purity and demonstrated improved HER electrocatalytic activity in an alkaline medium and excellent capacitance behavior. The NiS-4h electrode achieved the maximum HER activity with an overpotential of −159 mV vs RHE @ 10 Ag^−1^, Tafel slope of 218 mV dec^−1^, and long-term stability of −250 mV vs RHE @14 Ag^−1^, respectively. The same electrode demonstrated the highest capacitance of 3761 Fg^−1^ @10 mVs^−1^. Likewise, the PLI process with optimized laser parameters (532 nm, 10 Hz, 5 ns, 80 mJ laser pulse energy, and 15 min irradiation time) was used to prepare a copper-tin-zinc sulfide (CZTS) QDs with excellent purity^166^. The size of the CZTS QDs can be controlled by choosing suitable laser pulse energy. The as-prepared CZTS QDs-based cells provide maximum efficiency of 1.438% in dye-sensitized solar cells.

A few reports on the PLI synthesis of metal selenide and telluride have also been reported. Je et al. investigated the effect of various surfactants, such as CTAB, SDS, and PVP, on the formation of PbSe quantum dots using Pb and Se powder precursors^167^. The wavelength of 532 nm, a repetition rate of 10 Hz, and a duration of 7 ns were fixed as the laser parameters. The obtained rock-crystalline PbSe QDs possessed an average particle size of 6.83 nm. Gnatyuk et al. also constructed highly resistive p-type CdTe and CdZnTe single-crystals through the PLI process and studied their electrical and photoelectric behaviors^168^. They utilized a 694-nm ruby laser pulse to irradiate the sample for 20 ns at 300 K. The resultant In/CdZnTe/Au diodes offered excellent electrical and photoelectric properties with extremely low current leakage, making them promising candidates for X-ray and γ-ray detectors. The laser source, solvent, fluence, and irradiation time for the PLI-processed metal NPs, oxides, and nonoxide materials are tabulated in Table 5.

**Table 5:** Various laser, physicochemical, and morphological parameters, including laser source, solvent, fluence, and irradiation time, of PLI-processed metal NPs, oxides, and nonoxide materials

No.	Starting materials	Products	Laser source	Condition	Irradiation pulse/time	Particle size (nm)	Morphology	Ref.
1	Colloidal Au-NP obtained by chemical reduction	Au-NPs	Nd:YAG	*λ* = 532 nm; 10 Hz; 60 mJ pulse^−1^	10 ns	<20	Spherical	^132^
2	1 mM HAuCl_4_.3H_2_O	Lignin-functionalized Au-NPs	Quanta ray	*λ* = 532 nm; 10 ns; 10 Hz	10 min	15.67 ± 1	Spherical	^14^
3	Cu-NPs	Cu-submicrospheres	KrF excimer	*λ* = 248 nm; 1–10 Hz; 300 mJ pulse^−1^ cm^−2^	25 ns	100–322 (diameter)	Spherical	^136^
4	Cobalt carbonyl	Co-NPs	Nd:YAG	*λ* = 355, 266 nm; focal length ~5 mm; 1–10 Hz; 15 ± 3 mJ pulse^−1^ cm^−2^	30 min	<5	Spherical	^137^
5	Au/SiO_2_/Si (obtained by PVD)	Au/Si nanocomposite	Nd:YAG	*λ* = 532 nm; 2 Hz; 1.24 kJm^−2^. Beam diameter 6 mm	5–7 ns	<20	Spherical	^141^
6	Silver trifluoroacetate, Au(III) chloride trihydrate in ethylene glycol	Au-Ag nanocages	Nd:YAG	*λ* = 750 nm; repetition rate ~10 Hz; 30 mJ pulse^−1^ cm^−2^	5–7 ns	4–6	Nanocages	^131^
7	Au-seed solution/Ag plate in PVP	Au-Ag-NPs	Nd:YAG	*λ* = 1064 nm; repetition rate~10 Hz; 30 mJ pulse^−1^ cm^−2^	10 ns	8 ± 0.83	Spherical	^240^
8	Colloidal Au-solution and Co-oxide (60:40)	Au-Co-NPs	Nd: YAG (second-harmonic unfocused)	*λ* = 532 nm; repetition rate~30 Hz; 100 mJ pulse^−1^ cm^−2^	1 h	5–10	Sub-microsphere	^140^
9	1 mm Cu and Pd in ethanol	Cu_0.8_Pd_0.2_ NPs	Nd:YAG	*λ* = 532 nm; repetition rate ~10 Hz; 0.37 J pulse^−1^ cm^−2^; 150 mm focal length	5 ns	<10	Spherical	^142^
10	PLA of colloidal Ni solution followed by PdCl_2_ addition in methanol	Ni-Pd-NPs	Nd:YAG	*λ* = 532 nm; repetition rate~10 Hz; 80 mJ pulse^−1^ cm^−2^;	10 ns	16.83 ± 0.5036	Microspheres	^1^
11	Commercial EGaIn alloy	Ga-In core-shell	Nd:YAG	*λ* = 1064 nm; repetition rate ~10 Hz; pulse width 10 ns; 50–150 mJ pulse^−1^ cm^−2^; 9 mm beam diameter	5 min	9.49 nm	Quantum dots	^143^
12	ZnO powder in isopropyl alcohol	ZnO NPs	Nd:YAG	*λ* = 532 nm; repetition rate ~10 Hz; pulse width 10 ns; 280 mJ pulse^−1^; 9 mm beam diameter	10 ns for 60 min	<100	Irregular spherical	^153^
13	ZnO nanowires obtained by hydrothermal process	ZnO NPs	KrF excimer	*λ* = 248 nm; repetition rate ~10 Hz; pulse width 10 ns; 210 mJ pulse^−1^; focal distance 50 cm	25 ns	3.6 μm	Spherical	^152^
14	Colloidal CuO solution (acetone)	CuO-NPs	Nd:YAG	*λ* = 355, 532, and 1064 nm; repetition rate ~10 Hz; pulse width 10 ns; 133 mJ pulse^−1^	60 s/10 min	34	Spherical	^145^
15	White TiO_2_ powder	Black TiO_2_ NPs	Nd:YAG	*λ* = 532 nm; repetition rate ~10 Hz; pulse width 10 ns; 0.65 J cm^−2^	10 ns/45–90 min	<14	Rectangular shape	^147^
16	Ferrous and ferric chloride in NaOH	Fe_3_O_4_-FeO composite	Nd:YAG (third harmonic)	*λ* = 355 nm; repetition rate ~30 Hz; pulse width 10 ns; 33–177 mJ pulse^−1^ cm^−2^	1 h	6	Spherical	^144^
17	Lanthanum nitrate and titanium butoxide in water (La/Ti ratio = 0.4%)	La-doped TiO_2_ nanorod	Nd:YAG	*λ* = 532 nm; repetition rate ~200 Hz; pulse power 113 MWcm^−2^;	10 ns	101 ± 28	Nanorod	^149^
18	CuO and Au-NPs obtained by PLA in water	Au-CuO composite	Q-switched Nd:YAG	*λ* = 1064 nm; repetition rate ~5 Hz; focal length 80 mm; 1.5 mJ pulse^−1^ cm^−2^	10 ns	9.7–18.5	Spherical (irregular)	^159^
19	PMMA, zinc acetate in propanol (ultrasonication)	ZnO-PMMA composite	Nd:YAG	*λ* = 532 nm; repetition rate ~10 Hz; focal length 80 mm; 200 mJ pulse^−1^ cm^−2^	6 ns	9 ± 5	Uniform spherical	^155^
20	NiO in ethyl acetate	NiO-Ni	Nd:YAG	*λ* = 532 nm; repetition rate ~10 Hz; 130–520 mJ pulse^−1^ cm^−2^	10–90 min	360 ± 50	Sub-micrometer spheres	^161^
21	NiS solution (mechanical agitation of thiourea and nickel acetate)	NiS	Nd:YAG	*λ* = 1064 nm; repetition rate ~10 Hz; pulse width 4–6 ns; 700 mJ pulse^−1^ cm^−2^	1 h	40–60	Nanosheets	^165^
22	NaBH_4_ reduction of lead nitrate and selenium chloride in water	PbSe	Nd:YAG	*λ* = 355, 532, and 1064 nm; repetition rate ~10 Hz; pulse diameter 7 mm; 20–80 mJ pulse^−1^	7 ns	<10	Quantum dots	^167^

Halides, sulfides, selenides, and tellurides are highly carcinogenic and would cause ecological pollution. Hence, the growth of metal halide (MX) NPs with specific shapes and sizes (<5 nm) is a challenging task, which could be rectified using the simple and effective PLIL method. The above discussions prove that the MX NPs with the desired shape (mostly spherical) and size (5–10 nm) could be achieved using specific laser fluence, energy, and solvent.

#### Carbon and carbide materials

The advancement in the synthesis processes of 2D and mesoporous-based materials has permitted the integration of carbon-based materials in electronics, energy, biomedicine, and environmental fields^169^. Among the advanced synthesis techniques, PLI becomes an inevitable one due to the high purity and unique physiochemical properties obtained. In the early 2000s, Hu et al. constructed functionalized carbon NPs by irradiating graphite powder in a series of solvents, including diethanolamine, diamine hydrate, and polyethylene glycol using an Nd:YAG laser with a pulse power and wavelength of 6 × 106 W cm^−2^ and 1.064 μm, respectively^170^. By varying the solvents, the surface ligands and luminescent properties can be changed. The resultant black carbon NPs displayed a quantum yield of 0.05 with excellent integrated emission intensity (9.17). Likewise, CNPs and carbon dots have been generated by irradiating toluene using a nonfocused Nd:YAG pulsed laser^171^. The laser parameters such as pulse width, wavelength, repetition rate, and beam diameter were fixed as 8 ns, 1064 nm, 10 Hz, and 8 mm, respectively, for the carbon dot synthesis. The obtained carbon dots delivered enhanced photoluminescent characteristics. Recently, PEDOT-derived conducting polymer nanowires were synthesized through PLI, which showed improved sensing suitable for ultrasensitive photosensors^172^.

In the case of carbide nanomaterials, Ishikawa et al. investigated the laser irradiation synthesis of spherical boron carbide (B_4_C) from boron NPs in ethyl acetate solution, which involved melting by laser fluence followed by sudden quenching^173^. At optimized laser parameters, titanium carbide with improved surface properties (wear and erosion resistance) was developed by irradiating graphite-coated titanium sheets^174^. Interestingly, the prepared TiC showed a 10-fold enhancement in hardness (1700 HV) compared to that of the titanium substrate (160 HV). Besides, a series of carbon-encapsulated magnetic NPs, such as Ni–C, Fe–C, and Co–C, have been prepared by PLI in solution by Park et al., which displayed improved magnetic susceptibility^175^. This process paves a way for the fabrication of spherical submicron ceramic particles (carbides, nitrides) in a facile and efficient manner.

Overall, the section “Photo- and electrocatalytic materials: an overview” explains the outline and salient features of the family of photo/electrocatalytic materials and their key properties, such as laser parameters, solvents, shape, and size with appropriate literature (Tables 3–6). The PLAL syntheses of metal NPs and their composites have been used for catalyst applications especially for photo- and electrocatalytic processes, owing to their characteristics, including micron and submicron particles, various morphological shapes, distinct SPR, and photoluminescent characteristics. Thorough explanations of the state-of-the-art with the wide application of laser-assisted materials to photocatalysis to ORR of electrocatalytic systems are provided (section “Applications of laser-assisted materials”). The section “Applications of laser-assisted materials” deals with the basic mechanism of photo- and electrocatalytic process and the necessity of PLAL/PLIL-derived nanocatalysts, using recent literature, is discussed elaborately in detail.

**Table 6:** Various photocatalytic materials produced by laser-assisted techniques for the degradation of toxic pollutants

No.	Material	Morphology	Synthesis method	Solvent used	Laser parameters	Application	Efficiency	Ref.
1	ZnO-MWCNTs	Tubes and particles	PLAL	H_2_O (functionalized CNT solution)	Nd:YAG laser, *λ* = 1064 nm, 7 ns, 10 Hz, 100 mJ-Metal: Zn metal	Methylene blue (MB)	80%	^241^
2	Oxygen-deficient black-ZnO	Spherical	PLAL	Isopropanol	Nd: YAG laser, *λ* = 532 nm, 10 ns, 10 Hz, Metal: Zn powder	Rhodamine B (RhB)	90%	^153^
3	ZnO-Ag nanoparticles	Spherical	PLAL	H_2_O	Laser 1064 nm, 30 min, Metal: Zn plate, Ag plate	Rhodamine 6G (R6G)	90%	^242^
4	MWCNTs/Ag nanocomposite	Tubes and particles	PLAL	H_2_O (functionalized MWCNTs solution)	Nd:YAG laser, *λ* = 1064 nm, 7 ns, 10 Hz, 100 mJ- Metal: Ag-metal	4-Nitrophenol (4-NP), MB, methyl orange (MO)	90%, 100%, 100%	^243^
5	Ag metallic nanoparticles (NPs) and Ag/Au nanocomposites	Spherical	PLAL	H_2_O and chloroauric acid	Nd:YAG, *λ* = 1064 nm, 7 ns, 10 Hz and 60 mJ, Metal: Ag plate	4-NP	100%	^244^
6	Plasmonic ZnO/Au/g-C_3_N_4_ nanocomposites	Spherical and sheets	PLI	H_2_O and methanolic solution of HAuCl_4_·3H_2_O	Q-switched Nd:YAG, *λ* = 1064 nm, 7 ns, Metal: Zn plate	MB	100%	^245^
7	Copper NPs	Spherical, rod-like, cubes	PLAL	H_2_O, methanol, ethanol, 1-propanol, butanol, ethylene glycol, hexane, and acetonitrile	Q-switched Nd:YAG, *λ* = 1064 nm, 7 ns, 80 mJ pulse^−1^, Metal: Cu-plate	4-NP and nitrobenzene	100%	^10^
8	ZnO:Au nanocomposites	Spherical	PLAL	ZnO colloid solution and HAuCl_4_.4H_2_O	Q-switched Nd:YAG, *λ* = 532 nm, 5 ns, 10 Hz, 110 mJ pulse^−1^, Metal: Zinc powder	Rh B	70%	^246^
9	Ag/TiO nanoparticles in PVDF membrane	Spherical	PLA	H_2_O	Femtosecond laser, *λ* = 1030 nm, 400 fs, 1 MHz, Metal: Ti foil	Oil–water separation	97%	^247^
10	WO_3_/MWCNTs	Spherical and tubes	PLAL	H_2_O (functionalized MWCNT)	Q-switched Nd:YAG, *λ* = 1064 nm, 7 ns, 10 Hz, 80 mJ pulse^−1^, Metal: Tungsten	4-NP	100%	^248^
11	PVA/AgNPs	Spherical	PLAL	PVA solution	Nd:YAG, *λ* = 1064 nm, 7 ns, 10 Hz, 75 mJ pulse^−1^, Metal: Ag plate	4-NP	100%	^249^
12	g-C_3_N_4_–CdS	Spherical and sheets	PLAL	H_2_O	Nd:YAG, *λ* = 532 nm, 9 ns, 10 Hz, 350 mJ pulse^−1^, Metal: g-C_3_N_4_–CdS mixture	MB and Rh B	100% and 60%	^250^
13	TiO_2_ core-shell microspheres	Spherical	PLAL	H_2_O	Nd:YAG, *λ* = 355 nm, 3.6 ns, 20 Hz, 120 mJ pulse^−1^, Metal: Aqueous TiO_2_ powder	MB	99%	^251^
14	WO_3_-rGO	Spherical and sheets	PLAL	H_2_O	*λ* = 355 nm, 0.8 nm, 280 mJ pulse^−1^, Metal: aqueous solution of WO_3_	MB	78%	^192^
15	WO_3_-nCdS	Spherical and sheets	PLAL	H_2_O	Nd:YAG, *λ* = 532 nm, 5 ns, 10 Hz, 350 mJ pulse^−1^, Metal: WO_3_-CdS mixture	MB	100%	^196^
16	ZnO/Au/Pd	Spherical	PLAL	Methanol	Q-switched Nd:YAG, *λ* = 1064 nm, 7 ns, 10 Hz, 90 mJ pulse^−1^, Metal: Zn metal plate	MB	100%	^252^
17	FeS particles	Needle-like/sheet-like	PLI	H_2_O or ethanol	Nd:YAG, *λ* = 355 nm, 6 ns, 10 Hz, Metal: FeS pellet	MB	73%	^253^
18	CeO_2_/Ce_2_O_3_ nanohybrid	Spherical	PLAL	H_2_O	Nd:YAG, *λ* = 355 nm, 10 ns, 10 Hz, 180 mJ pulse^−1^, Metal: aqueous CeO_2_	MB	25%	^193^
19	Ag/ZnO	Spherical	PLAL	Ethanol	Nd:YAG, *λ* = 532 nm, 8 ns, 10 Hz, 180 mJ pulse^−1^, Metal: ethanolic mixture of Ag and ZnO	MB	100%	^254^
20	Ag nanocluster	Spherical	PLAL	H_2_O	120 fs pulsed laser, Metal: Ag disk in water	MB	79%	^197^
21	ZnO nanostructure	Particles/rods/flowers	PLAL-H	H_2_O	Nd:YAG, *λ* = 532 nm, 8 ns, 10 Hz, Metal: metallic Zn plate	MB	97.4%	^255^
22	SiC–TiO_2_ nanocomposite	Irregular	PLAL	H_2_O	Nd:YAG, *λ* = 532 nm, 6 ns, 10 Hz, 350 mJ pulse^−1^, Metal: TiO_2_ and SiC nanoparticles	MO	77%	^188^
23	ZnO/TiO_2_ nanocomposite	Spherical particles and rods	PLAL	H_2_O	Nd:YAG, *λ* = 532 nm, 6 ns, 10 Hz, 350 mJ pulse^−1^, Metal: TiO_2_ nanoparticles and ZnO nanorods	MO	100%	^182^
24	TiO_2_/CdS nanocomposite	Spherical particles	PLAL	H_2_O	Nd:YAG, *λ* = 532 nm, 8 ns, 10 Hz, 350 mJ pulse^−1^, Metal: TiO_2_ and CdS mixture	MO	82%	^186^
25	Au–CoFe_2_O_4_ and Au–SrTiO_3_	Spherical	PLAL	H_2_O	KrF (k = 248 nm) excimer laser, Metal: CoFe _2_O _4_ and SrTiO _3_	Rh B and MO	60% and 80%	^256^
26	SnO_*x*_ nanoparticles	Spherical	PLAL	H_2_O	Nd:YAG, *λ* = 1064 nm, 6 ns, 10 Hz, 100 mJ pulse^−1^, Metal: SnOx nanoparticles	MO and potassium dichromate	100% and 100%	^257^
27	MoS_2_/Black TiO_2_	Spherical and sheets	PLAL	H_2_O	Nd:YAG, *λ* = 355 nm, 3.6 ns, 20 Hz, 25 mJ pulse^−1^, Metal: TiO_2_ powder	Arsenite photooxidation	96%	^189^
28	TiO_2_–ZSM5–MoS_2,_	Spherical and sheets	PLAL and microwave	H_2_O	Nd:YAG, *λ* = 1064 nm, 3.6 ns, 20 Hz, 180 mJ pulse^−1^, Metal: MoS_2_ powder	Arsenite oxidation	100%	^190^
29	Au-Pd@g-C_3_N_4_	Spherical and sheets	PLAL	H_2_O	Nd:YAG, *λ* = 1064 nm, 300 mJ pulse^−1^, Metal: gold plate and aqueous PdCl_2_	Benzene-to-Phenol Conversion	98%	^258^
30	Cu-20 min/a-C_3_N_4_	Spherical and sheets	PLAL	H_2_O	Q-switched Nd:YAG, *λ* = 1064 nm, 5 ns, 350 mJ pulse^–1^, Metal: Copper	Oxidation of cyclohexane to the ketone-alcohol	88%	^259^

## Applications of laser-assisted materials

### Photocatalysis

PLAL is used for preparing potential photocatalysts to degrade various toxic compounds, such as MB, rhodamine 6G (R6G), rhodamine B (RhB), 4-nitrophenol, methyl orange, and nitrobenzene. This section discusses the photocatalytic applications of the materials produced via pulsed laser techniques. Engineering of materials through the PLIL considerably reduces particle size with a narrow size distribution, and no surfactants are required. Laser-synthesized materials exhibit a clean surface without the blocking effect of chemical precursors and molecular ligands. In addition, laser irradiation generates high temperature and pressure locally, followed by fast cooling of the products. These preparation processes incorporate defects and disorders in structure, resulting in decreased particle size and electronic state modification.

**Fig. 15:**
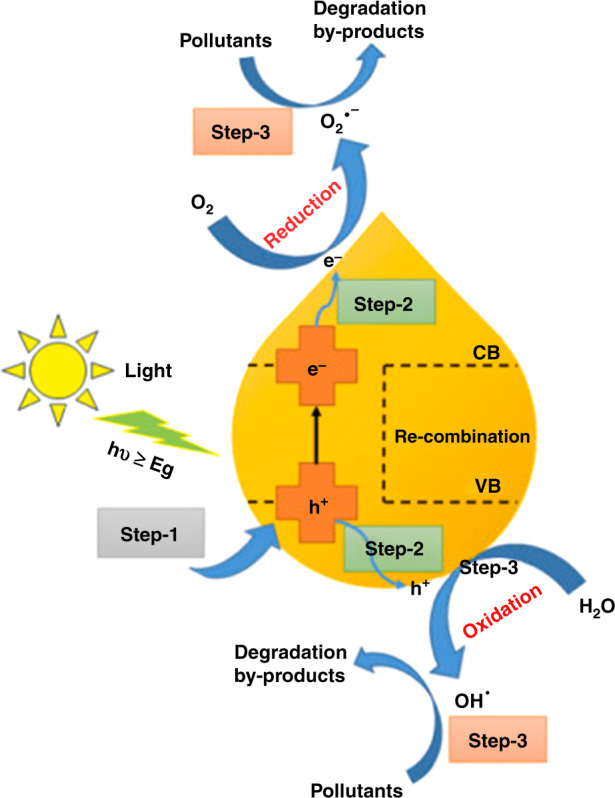
Mechanistic illustration of photocatalytic degradation involved in the degradation of organic molecules^178^. Copyright (2021) Elsevier

Photocatalysis involves the use of light energy to drive chemical reactions. The mechanism of photocatalysis in a semiconductor material involves the absorption of photon energy (visible-light-active semiconductor can absorb solar energy), which is equal to or greater than the bandgap energy of the photocatalyst, resulting in the formation of electron and hole pairs as charge carriers. These charge carriers are involved in various oxidation and reduction reactions on the photocatalyst surface. The electron reacts with dissolved oxygen leading to the formation of superoxide radical anions (O_2_^−•^), which result in hydroxyl (OH^•^) radical production. These OH^•^ radicals are responsible for the degradation of organic compounds^176,177^. There are three major steps involved in the photocatalytic mechanism: (1) photoinduced separation of electron-hole (e^−^-h^+^) charge carriers (e^−^ at the conduction band and h^+^ at the valence band), (2) distribution of charge carriers on the photocatalyst surface, and (3) scattered charge carriers undergoing reduction and oxidation reactions to produce various free radicals, which are responsible for the degradation of organic molecules, as depicted in Fig. 15^178^. The pulsed laser synthesis process is used for the synthesis of potential photocatalysts, which are active in the degradation of various toxic compounds, such as MB, R6G, RhB, 4-nitrophenol, MO, and nitrobenzene. This section discusses the photocatalytic applications of the materials produced via pulsed laser techniques.

#### ZnO-based materials

**Fig. 16:**
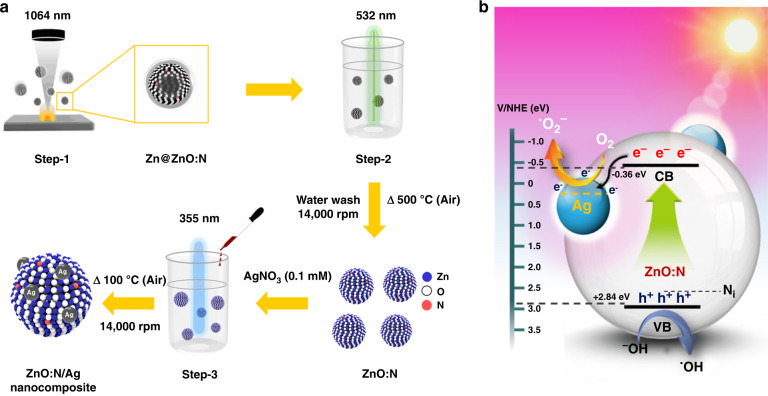
PLA and PLI process for ZnO based composites. **a** Schematic illustration of the fabrication of ZnO:N/Ag via two-step PLA and PLI process, and **b** Photocatalytic degradation mechanism of RhB over ZnO:N/Ag catalyst under solar light^185^. Copyright (2021) Elsevier

Yang et al. prepared cubic phase ZnO_2_ (zinc peroxide) NPs through PLAL using a 532-nm nanosecond pulse laser in H_2_O_2_ as the ablation media and Zn powder as the solid target. The laser energy and ablation time both influenced the ZnO_2_ formation. ZnO_2_ exhibited efficient photocatalytic degradation activity for the removal of RhB, which could be attributed to the indirect bandgap of 3.4 eV and comparatively positive VB edge potential; in addition, the hydrophilic wetting behavior of ZnO_2_ contributed to better interfacial interactions between ZnO_2_ and hydrophilic RB molecule, resulting in the higher photocatalytic activity of ZnO_2_^179^. Moqbel et al.^180^ reported ZnO/rGO nanocomposites fabricated via PLAL and employed them as efficient photocatalysts for the degradation of RhB as a targeted pollutant under visible light irradiation (VLI). Anchoring of ZnO on rGO nanosheets in ZnO/rGO nanocomposite significantly reduced the aggregation of ZnO and improved the surface area, resulting in more adsorption of reactants on the surface, enhanced light absorption in the visible spectral region, and rGO contributing to faster charge carrier transport across the surface, which resulted in 86% of RhB degradation compared to ZnO ~40%. Blažeka et al.^181^ reported the PLA-assisted synthesis of ZnO NPs and studied the photocatalytic degradation of RhB and MB. The photocatalytic degradation efficiency of around 40% was higher for MB compared to RhB due to the stronger UV light absorption of MB than RhB. Gondal et al.^182^ reported the integration of ZnO nanorods with TiO_2_ via PLAL using an Nd:YAG laser at 532 nm wavelength and studied photocatalytic and photovoltaic performances. The ZnO/TiO_2_ (9:1) nanocomposite showed 98% degradation performance for MO and high photon conversion efficiency of 6.7% in DSSC (dye-sensitized solar cells), which might be due to the presence of oxygen vacancy defects that acts as trapping sites for charge carriers, resulting in decreased electron-hole recombination. Mintcheva et al.^183^ prepared ZnO nanospheres and nanorods through the laser ablation of Zn metal plate and investigated photocatalytic activity for MB oxidation. The nanorods formed with millisecond laser exhibited higher photocatalytic activity than the spherical nanostructures obtained by nanosecond laser due to a larger number of defects on ZnO nanorods. Recently, our research group also focused on the development of various photocatalytic materials using pulsed laser-assisted techniques and employed them for the wastewater remediation process. For example, Naik et al.^184^, reported rGO supported ZnO/Au/rGO nanostructure prepared via a two-step process, such as PLAL and wet chemical impregnation process. The optimized ZnO/Au15/rGO5 ternary nanocomposite showed higher MB degradation efficiency of 95% in 120 min under solar light irradiation, which was attributed to the improved light absorption, enhanced charge separation, and migration by the anchored Au and rGO materials on ZnO. In another report by our group, metal (Ag) and nonmetal (N) co-doped ZnO were fabricated and their RhB degradation performances were evaluated (Fig. 16a, b)^185^. The co-doped ZnO:N/Ag material exhibited six times enhanced activity than that of pure ZnO, which was attributed to better light absorption and reduced recombination of charge carriers. Kanakkillam et al.^153^ prepared oxygen-deficient defect-rich black-ZnO by PLI in liquids and observed that the bandgap decreased with the increase in PLI time. The black-ZnO was studied for RhB dye degradation and achieved ~90% degradation due to the improved visible light absorption on defect-rich ZnO.

#### TiO_2_-based materials

Ilyas et al.^186^ reported the preparation of TiO_2_/CdS composite using PLAL process using 532 nm wavelength using an Nd:YAG laser. CdS was introduced as a composite material to decrease the bandgap and enhance visible light absorption. The composite activity was studied in DSSCs and photocatalytic MO degradation. Ten wt.% CdS-incorporated TiO_2_ exhibited better activity than that of pure TiO_2_ with an improved power conversion efficiency from 0.6% to 4.3%, and 82% of MO degradation efficiency in 36 min under VLI. The enhanced activity was ascribed to the reduced electron-hole recombination, increased charge conduction, longer lifetime of charge carriers, and reduced photocorrosion.

Liu et al.^187^ reported rutile TiO_2_ prepared by PLAL in poly-(vinylpyrrolidone) solution and evaluated their photocatalytic activity for MB degradation. The produced rutile TiO_2_ with controlled size exhibited an enhanced photocatalytic performance. Gondal et al.^188^ prepared SiC–TiO_2_ NPs through PLAL and evaluated for photocatalytic MO as targeted pollutant degradation and used as an efficient photoanode in DSSCs. The incorporation of SiC enhanced the catalytic activity by improving the MO degradation efficiency by about 77%, and the photon conversion efficiency of fabricated DSSC was enhanced from 0.6% to 1.65%, which was attributed to the improved electron transfer ability of SiC. Similarly, the same group fabricated ZnO/TiO_2_ nanocomposite and employed them as an efficient photocatalyst for MO degradation and as a photoelectrode in DSSCs^182^. Balati et al.^189^ reported MoS_2_ nanosheets vertically aligned on black TiO_2_ (MoS_2_/B-TiO_2_) (consisting of rutile and hydrogenated anatase TiO_2_) synthesized through PLAL. The MoS_2_/B–TiO_2_ depicted high arsenite photooxidation of 96.6% compared to that of B-TiO_2_ (70.3%) and arsenate adsorption efficiency of 5200 μg g^−1^ compared to that of B-TiO_2_ (1600 μg g^−1^), which was attributed to the extended visible light absorption in the composite along with improved charge carrier accumulation and separation. The same research group also fabricated ternary catalyst TiO_2_–ZSM5–MoS_2,_ which exhibited 100% arsenite photoconversion to arsenate owing to the structural defects of the ternary catalyst and improved electrical conductivity caused by vertically aligned MoS_2_ on TiO_2_^190^.

Environmentally friendly and facile pulsed laser process was used to prepare TiO_2_, TiO_2_/CdS, Ag/TiO_2_, SiC-TiO_2_, ZnO-TiO_2_, MoS_2_/Black TiO_2_, and TiO_2_-ZSM5-MoS_2_ nanocomposites, which exhibit properties, like decreased electron-hole recombination, increased charge conduction, longer charge carriers lifetime, high charge carrier accumulation, and reduced photocorrosion. Ag/TiO_2_ prepared through conventional methods like photoreduction shows the drawback of weak Ag deposition on TiO_2_ surface, which can be improved by using pulsed laser synthesis.

#### Graphene oxide-based materials

Chen et al.^191^ reported the synthesis of graphene (Gr)/Au and GO/Au nanocomposites through pulsed laser-induced photolysis in the presence of hydrogen peroxide, chloroauric acid (HAuCl_4_) along with Gr or GO in an aqueous solution. The nanocomposites were evaluated for MB degradation, where both Gr/Au and GO/Au exhibited 92% and 94% removal efficiency due to reduced recombination of electron-hole. The Schottky barrier formed between the Au–Gr interface due to the higher work function of Au than Gr. The electron injected on Gr rapidly moved to Au-NPs, leading to the spatial separation between MB^+^ and electrons, thus reducing the recombination of charge carriers. The decreased concentration of electrons on the Gr surface predominantly increases the electron transfer from MB^−^ to Gr and the surface-adsorbed O_2_ traps the electron from Au, leading to the formation of several oxidative species (OH^−^) because Au is a potential electron donor; hence, it shows the improved photocatalytic performances of the catalysts. Ibrahim et al.^192^ reported WO_3_ NPs anchored on rGO sheets for photocatalytic degradation and energy storage application. WO_3_-rGO shows high catalytic activity for MB degradation and energy storage with a specific capacitance of 577 F g^−1^, power density of 1000 W kg^−1^, and energy density of 76.3 Wh Kg^−1^. Gr/Au and GO/Au composites prepared via a pulsed laser process exhibited high activity attributed to reduced recombination of e^−^/h^+^. WO_3_/rGO composites prepared through laser irradiation involved a simultaneous reduction of GO to rGO and WO_3_ nanostructure anchoring on rGO, thereby reducing preparation time and additional chemical utilization for reduction.

#### CeO_2_-based materials

Ma et al.^193^ prepared CeO_2_/Ce_2_O_3_ nanohybrids using PLAL and studied the photocatalytic evaluation for MB degradation. The hybrid CeO_2_/Ce_2_O_3_ showed higher photocatalytic activity compared to CeO_2_, which might be due to the oxygen vacancy-induced Z-scheme process, which efficiently separates charge carriers, resulting in improved photocatalytic performance. Green synthesis of CeO_2_ nanoparticles via pulsed lasers showed high activity ascribed to well-separated and defect-rich particles. Hence, these syntheses can easily be further transformed into large-scale production of various nanostructured materials.

#### Cu-based materials

Li et al.^194^ reported Cu_2_O-Cu nanocomposite synthesized using low-power CO_2_ laser and studied its photocatalytic activity for the degradation of MB. The particle size can be controlled by tailoring the laser energy and NaOH concentration. The photocatalytic activity was compared with hydrothermally synthesized Cu_2_O-Cu (H-Cu_2_O-Cu), among which the laser-assisted nanocomposite showed better activity than the hydrothermally synthesized material with 90% MB degradation in 50 min, whereas H-Cu_2_O-Cu exhibited 51% efficiency toward MB degradation. The improved activity in the laser-induced method was due to the presence of a highly active Cu_2_O (111) facet, which has more Cu dangling keys, while H-Cu_2_O-Cu showed (110) and (100) facets. Cu-based nanocomposites without byproducts can be prepared through PLAL/PLIL methods. Cu_2_O-Cu using PLAL process exhibited higher activity compared with hydrothermally synthesized material Cu_2_O-Cu because of the high concentration of Cu_2_O (III) facet. Hence, facet engineering through pulsed laser preparation is an important field to explore. In addition, the use of different solvents for PLI results in the formation of different sizes, morphology, and properties of materials.

#### CdS-based materials

Moqbel et al.^195^ reported the PLAL synthesis of CdS/RGO nanocomposite and studied photocatalytic degradation efficiency toward the removal of MB. The optimized concentration of 5% RGO in CdS/RGO composite showed higher degradation efficiency of 77%, which was 2.65 times greater than that of CdS, and the enhanced results might be due to the reduced charge recombination process and synergistic effect between CdS and RGO. Hassan et al.^196^ prepared WO_3_-CdS composite using PLAL and showed complete MB degradation. In addition, they employed it as an electrode material for a supercapacitor, which depicted a high specific capacitance of 68.1 mF cm^−2^ equivalent to 121.2 Fg^−1^ with a large energy density of 7.7 and 254 μW cm^−2^.

#### Ag-based materials

Santillán et al.^197^ prepared fluorescent Ag nanoclusters using ultrashort light pulses by PLAL. The Ag-NPs exhibited small size with few atoms, resulting in excellent photocatalytic activity for MB degradation with an efficiency of 79%. In general, pure Ag and GO compounds are excellent support materials/cocatalysts for photo- and electrocatalytic applications. The pulsed laser process generates ions and atoms in the plasma plume, which reaches different nucleation stages with varied sizes from large (>20 nm) to small nanoparticles (<1 nm). Table 6 shows the various photocatalytic materials produced by laser-assisted techniques for the degradation of toxic pollutants.

### Electrocatalytic hydrogen evolution

The increasing population and economic growth increase the energy demand. Presently, 90% of global energy is produced from fossil fuels, although fossil fuels are non-renewable and release toxic CO_2_ gases into the environment, leading to global climate change. Yet, the energy demand is projected to double by mid-century and more than triple by 2100. Therefore, providing clean and sustainable energy is an urgent issue. Hydrogen fuel is an ideal solution to tackle the energy crisis^198^ and is a sustainable, energy-dense, and eco-friendly clean fuel material that can be produced even via water splitting^199^. Therefore, an easy and efficient process for hydrogen production is necessary. Presently, commercial hydrogen is produced from various techniques, such as cryogenic distillation, gasification of coal, steam reforming of natural gas, and water splitting. Among all, hydrogen evolution through electrocatalytic water splitting is more economical and environmentally friendly because water splitting occurs at ambient temperature and pressure using water and less electricity, leading to reduced production costs for H_2_. The HER is a two-electron transfer reaction, and the rate of the reaction depends on ΔG_H_ (Gibbs free energy for hydrogen adsorption). HER reaction can be conducted in a hydrogen-saturated electrolyte at 1 atm to give a distinct, non-drifting reversible hydrogen potential. Pulsed laser synthesis is widely used to synthesize the electrocatalysts for HER, and the following section focuses on the electrocatalytic activity of the materials produced via pulsed laser techniques.

Li et al.^200^ prepared amorphous molybdenum sulfide (a-MoSx) using femtosecond laser ablation of precursor ammonium tetrathiomolybdate in an aqueous solution. The photoinduced oxidation/reduction can be controlled through pulse delay and by adjusting the pulse energy to modify the S-to-Mo ratio in a-MoS*x*. The optimized a-MoS*x* exhibited high electrocatalytic activity with an overpotential of 250 mV, Tafel slope of 40 mV dec^−1^, and large current density of 516 mA cm^−2^, which can be attributed to the bridging S_2_^2−^ ligands and Mo^V^ defect species. Chen et al.^201^ prepared RuAu single-atom alloy as a potential electrocatalyst for HER activity. RuAu was prepared through PLAL and exhibited a low overpotential of 24 mV@10 mA cm^−2^, which is lesser than that of the Pt/C catalyst (46 mV) in alkaline media along with high stability and high turnover frequency. The exceptionally high activity was ascribed to the synergistic catalytic effect of Ru and Au active sites.

Wang et al.^202^ prepared a porous carbon composite that involved Ru and nitrogen (Ru@CN) from biomass honey using the laser conversion method. The electrocatalytic activity of the Ru@CN was improved by altering the loading of Ru, N, and laser power. Thus, the optimized catalyst with 0.04-Ru@CN-6 with Ru loading amount of 2.66 wt.% showed higher activity in all pH conditions. The 0.04-Ru@CN-6 in alkaline media exhibited a reduced overpotential of 11 mV at 10 mAcm^−2^, which was lower than that of the commercial Pt/C (37 mV). The Ru@CN showed an overpotential of 45 and 30 mV in neutral and acidic solutions, respectively, and the improved activity was attributed to the presence of rich Ru^0^ and Ru-N sites. Ji et al.^203^ reported the one-step laser-assisted synthesis of W@WS_2_ core-shell nanospheres (CSNS) for electrochemical hydrogen evolution. The introduction of multifunctional CSNS contributes to the hierarchical curves of metallic WS_2_ on W core with a curve as high as 10^−2^ nm^−1^, making the WS_2_ interlayer spacing broader, exposing more catalytic sites. The W core improved the electrical conductivity by 4.5 times, and the WS_2_ electrochemical surface area was also enlarged. The CSNS optimizes the hydrogen adsorption Gibbs free energy and electronic occupation around the Fermi level. W@WS_2_ CSNSs exhibit significant electrocatalytic activity with a small overpotential of 161 mV at 10 mAcm^−2^, Tafel slope of 34.5 mV dec^−1^, and higher electrical double layer capacitance 62.2 mF cm^−2^, and W sites are the most catalytically active sites. Yuan et al.^204^ prepared Ni*@*N-doped carbon nanotubes through laser processing and gas–solid calcination as an efficient electrocatalyst for water splitting and photothermal conversion layer. The NiFe alloys foil (NiFe-L) was also prepared using laser ablation for oxygen evolution reaction electrode to develop a two-electrode electrolyzer for (–)Ni@NCNTs/NF-L/TE // NiFe-L (+) for overall water splitting. The HER analysis of Ni@NCNTs showed a lower overpotential of 81 mV at 10 mAcm^−2^, which was better than those of Ni (318 mV) and NiO/NF-L (220 mV) in 1 M KOH, and the overall water splitting gave a voltage of 1.943 V at 50 mAcm^−2^, which was larger than that of Pt/C // RuO_2_(+) (1.895 V) but at larger current density, the voltage was smaller than that of Pt/C // RuO_2._

Wang et al.^205^ prepared CoRu nanoalloy@N-doped graphene derived from Ru-ZIF-67 through laser engraving at atmospheric temperature and pressure. The CoRu@NG-x was evaluated for electrocatalytic hydrogen evolution activity, which showed 62, 52, and 88 mV at a current density of 10 mA cm^−2^ in 1.0 M KOH, 0.5 M H_2_SO_4_, and 1.0 M PBS solution for HER catalysis. The synergistic effect of Ru content of 0.18 wt.% in cobalt and N-doped graphene layers contributes to the improved activity by accelerated electron transport; in addition, Ru provides richer active sites for HER. Pradhan et al.^206^ prepared MoS_2_ QDs through the photo-exfoliation of solid MoS_2_ by PLA. MoS_2_ QDs with ~4, 2.9, and 6 nm sizes were prepared by varying ablation duration (5, 10, and 20 min, respectively) at a constant laser energy of 40 mJ with an ablation duration of 5 min. The prepared MoS_2_ QDs showed a high HER activity with a lower Tafel slope of 57 mV dec^−1^. Shang et al.^207^ prepared single-atom catalysts (SAC) by embedding nickel atoms into Ru metal via PLA, resulting in NiRu SAC with an atomic-level interface with improved catalytic activity. The NiRu SAC showed significantly low overpotential for HER of 17 mV at 10 mAcm^−2^ with high durability, which was superior to that of the conventional Pt/C catalyst. NiRu-0.2 SAC exhibited remarkable oxygen evolution activity of 210 mV at 10 mA cm^−2^ and a water-splitting ability of 1.5 V@10 mAcm^−2^. The higher activity of NiRu SAC was ascribed to the better interface construction between oxidized single nickel atom, and Ru/O atom lowers the energy barrier for water dissociation in addition to H adsorption through the interaction between the interfacial Ru and O atoms. Table 7 shows the various electrocatalytic materials produced by laser-assisted techniques for hydrogen production via water splitting.

**Table 7:** Various electrocatalytic materials produced by laser-assisted techniques for hydrogen production via water splitting

No.	Material	Morphology	Synthesis method	Solvent	Laser information	Overpotential (mA/cm^2^)	Tafel slope (mV/dec)	Ref.
1	Ni-Pd spheres	Spherical	PLI and sonochemical	Methanol	Nd:YAG laser, *λ* = 532 nm, 10 ns, 10 Hz, 80 mJ, Metal: methanolic solution of PdCl_2_ and Ni	387 mV at 1 mA/cm^2^	168	^1^
2	Ag-NPs	Spherical	PLAL	H_2_O	Nd:YAG laser, *λ* = 1064 nm, 7 ns, 10 Hz, Metal: silver	96 mV at 10 mA/cm^2^	76.1	^260^
3	a-MoS*x*	Sheets	PLAL	H_2_O	Ti:sapphire laser system, 50 fs, 800 nm, 1 kHz, Metal: aqueous ammonium tetrathiomolybdate ([NH_4_]_2_MoS_4_)	250 mV at 10 mA/cm^2^	40	^200^
4	Ag-NPs with stacking faults	Spherical	PLAL	H_2_O	Nd:YAG laser, *λ* = 1064 nm, 7 ns, 15 Hz, Metal: silver	32 mV at 10 mA/cm^2^	31	^261^
5	Rh NPs	Spherical	PLAL	H_2_O or ethanol	Pulsed laser, *λ* = 1064 nm, 6 ns, Metal: rhodium plate	57 mV at 10 mA/cm^2^	55	^262^
6	RuAu single-atom alloy	Spherical	PLAL	HAuCl_4_ aqueous solutions	Nd:YAG laser, *λ* = 1064 nm, 7 ns, 15 Hz, Metal: Ru target	24 mV at 10 mA/cm^2^	37	^201^
7	Ru@CN	Spherical and sheets	CO_2_ laser conversion	–	Laser power 6 W, scanning speed 100 mm s^−1^, Metal: honey powder	11 mV at 10 mA/cm^2^	48	^202^
8	W@WS_2_ CSNSs	Spherical	PLAL	–	KrF excimer laser, *λ* = 248 nm, energy density 280 mJ mm^−2^, 5 Hz, Metal: W and WS_2_ powder	161 mV at 10 mA/cm^2^	34.5	^203^
9	Ni@NCNTs	Tubes	PLAL	–	Fiber laser, *λ* = 1064 nm, Metal: Ni foil	81 mV at 10 mA/cm^2^	64.2	^204^
10	CoRu nanoalloy@N-doped graphene	Spherical particles and sheets	Laser engraving	Ethanol and methanol	6 W 50 mm/s, Metal: Ru-ZIF-67	52 mV at 10 mA/cm^2^	90	^205^
11	Graphene/Cu_*x*_O@Ni foam	Spherical particles and sheets	Laser transfer process	Ethyl alcohol	M-SOLV MSV-200 W laser, *λ* = 1070 nm, 2.5 ms, Metal: Cu film	149.6 mV at 10 mA/cm^2^	157	^263^
12	Ni(OH)_2_/Ni foam	Spherical particles and porous sheet	PLAL	H_2_O	Ti: Sapphire laser, *λ* = 800 nm, 1k Hz, Metal: Ni target	187 mV at 20 mA/cm^2^	82	^264^
13	MoS_2_ QDs	Spherical QDs	PLAL	H_2_O	Q-switched Nd:YAG laser, *λ* = 532 nm, 8 ns, 10 Hz, Metal: bulk MoS_2_	534 mV at 10 mA/cm^2^	57	^206^
14	NiRu SAC	Spherical	PLAL	H_2_O	Nanosecond laser, Metal: Ru in aqueous NiCl_2_	17 mV at 10 mA/cm^2^	27	^207^
15	VSe_2_	Nanosheets	PLAL	H_2_O	Nufern NuQ fiber laser, *λ* = 1064 nm, 100 ns, 30 kHz, Metal: VSe_2_ rock	250 mV	–	^234^
16	MoS_2_ quantum dots	Spherical	PLAL	H_2_O	Nd:YVO_4_, UV to infrared, 10 ps laser pulses, 2 MHz	187 mV	53 mV·dec^−1^	^265^

Based on the above reports, the amorphous MoS_2_ with controlled Mo and S ratio with pulse delay produces highly HER active MoS_2_ material. CoO exhibits Co vacancies contributing to high activity, and Ru@CN shows Ru^0^ and Ru-N sites, W@WS_2_ shows the high electrochemical surface area and conductivity. In addition, Ni@NCNTs, NiO/NF-L, CoRu@NG, graphene/Cu_*x*_O@Ni foam, MoS_2_ QDs, and NiRu SAC synthesized using PLAL process depict high HER activity owing to high surface area and defects.

### Photocatalytic hydrogen evolution

Z-scheme structured TiO_2_/rGO/g-C_3_N_4_ composite was synthesized using PLAL and utilized as efficient photocatalysts for hydrogen production by Ibrahim et al.^208^. The optimized composite with TiO_2_ to g-C_3_N_4_ of 2:4 and 1% rGO exhibited a hydrogen evolution of 32 ± 1 mmol g^−1^h^−1^, which was superior to g-C_3_N_4_, TiO_2,_ and TiO_2_/rGO photocatalysts. High activity was due to the strong interfacial bonding between TiO_2_, rGO, and g-C_3_N_4_ composite, and improved visible light absorption contributed by the high photoresponsiveness of rGO and g-C_3_N_4_ resulted in efficient charge separation and transfer of charge carriers. Johny et al.^209^ prepared SnS_2_ NPs through PLAL in acetone and isopropanol mixture and studied the effect of laser on particle size and morphology toward HER under UV-LED irradiation.

Bao et al.^210^ prepared CoO nanoparticles for overall water splitting with an H_2_:O_2_ ratio of 2:1 and solar-to-hydrogen efficiency of 5%. The high photocatalytic activity of CoO was attributed to the electronic difference between the nanocrystal CoO and micropowders. The flat band potential of the CoO nanocrystal was around 1 V, which was less than the micropowder, and this might be due to Co vacancies in the p-type oxygen-rich CoO nanoparticles. Highly pure and environmentally friendly photocatalysts prepared through pulsed laser methods, such as CdSe/TiO_2_, TiO_2_/rGO/g-C_3_N_4_, SnS_2_, and CoO_2_, exhibited high catalytic activity attributed to efficient transfer of charge carriers and charge separation.

### CO_2_ reduction

**Fig. 17:**
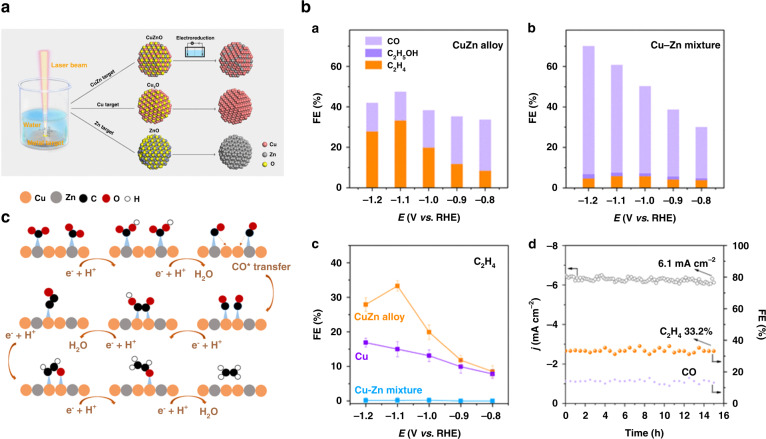
PLA assisted synthesis of CuZnO, ZnO, and Cu_2_O for electrochemical reduction of CO_2_. **a** Schematic design for the production of CuZnO, ZnO, and Cu_2_O using PLA in water followed by electroreduction. **b(**a–c) Faradaic efficiency (FE) values of the electroreduction of CO_2_ into value-added products over various catalysts, **b(**b–d) Current density and FE of CO and ethylene over CuZn alloy at –1.1 V vs RHE for 15 h, and **c** CO_2_ reduction mechanism on the CuZn NP surface^211^. Copyright (2021) American Chemical Society

The Ag- and Au-based materials are generally used as CO_2_ catalysts, which show high selectivity for CO formation, Zn-based catalysts produce CO, HCOOH, and syngas, Co- and Pb-based catalysts show high selectivity for formic acid, and Cu-based catalysts show high selectivity for hydrocarbons, such as methane, ethylene, and ethanol. Feng et al.^211^ reported CuZn electrocatalysts prepared via PLAL for CO_2_ reduction into ethylene. The CuZn alloy exhibits a high faradic efficiency value of 33.3% at a potential of –1.1 V Vs RHE, which was attributed to the higher concentration of Cu and Zn atoms on the catalyst surface-stabilizing CO* intermediate and facilitating the transfer of CO* from Zn atoms to Cu for further dimerization and protonation, resulting C_2_H_4_ product, as shown in Fig. 17.

Zhou et al.^212^ prepared PVP-based (100)-faceted Ag nanocubes anchored on rGO (Ag-rGO) through anisotropic growth by PLA. The cube-shaped Ag-rGO was tested for photocatalytic CO_2_ reduction into CO, which exhibited a conversion rate of 133.1 μmol g^−1^ h^−1^ in 6 h for the cubic Ag-rGO composite. rGO reduces the aggregation of Ag-NPs and improves electron mobility. In addition, the high concentration of PVP directs the formation of cubic morphology with a highly active (100) facet (low concentration of PVP forms spherical structure), contributing to high catalytic CO_2_ reduction by Ag-rGO nanocubes.

The pulsed laser process is adopted to prepare various homogeneous specific sizes and shape particles for CO_2_ reduction. Homogeneous element distributed CuZn catalyst shows high activity for CO_2_ electroreduction, and (100) facet-engineered Ag-rGO can be obtained via pulsed laser synthesis. Hence, specific facet-engineered nanoparticles can be prepared through the pulsed laser process.

### Oxygen evolution reaction

Water oxidation reaction plays a vital role in the production of clean energy but the kinetics associated with this reaction is slow. The reaction proceeds through either a four-electron or four-proton processes to generate O_2_. This section discusses the electrocatalytic materials prepared through PLA/PLI for OER applications.

**Fig. 18:**
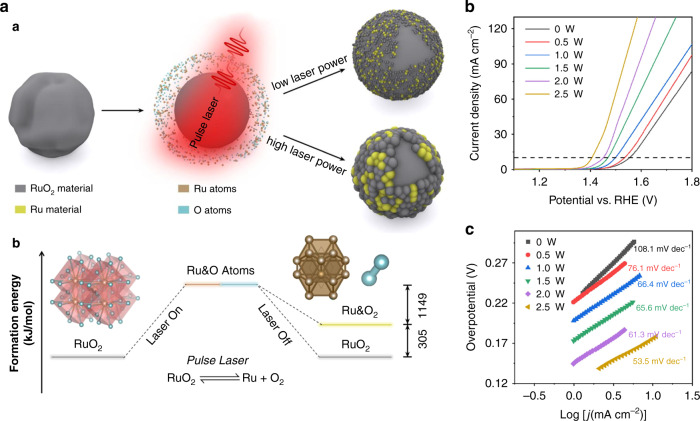
Ru based electrocatalysts productivity as a function of laser power. **a(**a, b) Schematic of Ru/RuO_2_ on RuO_2_ nanoparticle preparation using PLA, **b** LSV for OER, and **c** Tafel graph for OER^213^. Copyright (2021) Elsevier

Wang et al.^213^ prepared RuO_2_ NPs through PLA for OER in alkali and acidic media. The PLA treatment creates numerous Ru and RuO_2_ clusters on the surface of RuO_2_ NPs, forming a lychee-shaped morphology, as depicted in Fig. 18. The optimized RuO_2_ exhibited an overpotential of 172 mV at 10 mAcm^−2^ with a Tafel slope of 53.5 mV dec^−1^ in alkali medium (KOH) and 219 mV with a Tafel slope of 44.9 mV dec^−1^ in acidic medium (H_2_SO_4_). The improved OER activity was ascribed to the lychee-shaped shell, which enhances ECSA and electrical conductivity.

Abass et al.^214^ reported the green synthesis of rGO/GCN nanocomposite by irradiating GO/GCN dispersion in an aqueous methanol solution by a UV laser. The rGO/GCN composite was evaluated for photoelectrochemical water oxidation, which exhibited a nine-fold enhancement in current into ~90 mAcm^−2^ in alkaline media with a potential of 0.76 V vs RHE, which was attributed to the improved visible light absorption, reduced recombination, and excellent adhesion on the current collector. Marzun et al.^215^ prepared NiMo NPs through PLAL, which showed excellent activity for ORR without having any noble metals, such as Pt and Ir. The high activity was due to the dealloying of colloidal samples, i.e., dissolution of Mo contributing to the formation of a porous Ni surface, which could enhance the electrocatalytic behavior because of the removal of less noble components from the surface of a bimetallic alloy.

Wang et al.^216^ reported the preparation of iridium nanospheres (Ir NSs) with abundant atomic steps through PLAL. The Ir NSs exhibited high OER and HER activity in acidic media with a lower overpotential of 266 mV at 10 mA cm^−2^ with a Tafel slope of 58.7 mV dec^−1^ for OER, and 28 mV at 10 mAcm^−2^ with a Tafel slope of 17.8 mV dec^−1^ for HER. Ir NSs also showed overall water-splitting activity with a cell voltage of 1.535 V@10 mA cm^−2^, and the higher activity was attributed to the high chemically active Ir NSs because of the existence of numerous atomic steps with lower coordination numbers on the surface. Waag et al.^217^ reported structurally disordered CoFe_2_O_4_/CoO electrocatalysts prepared via pulsed laser fragmentation in liquid (PLFL) of CoFe_2_O_4_ in water. The CoFe_2_O_4_/CoO material showed a 23% decrease in overpotential for OER in aqueous alkaline media with a Tafel slope of 71 mV dec^−1^. The catalytic activity can be modified by varying the PLFL treatment cycles in addition to thermal melting, and decomposition during PLFL cycles was altered by laser beam energy. Thermal decomposition results in the reduction of particle size and crystal conversion to crystalline CoO and amorphous CoFe_2_O_4_. Waag et al.^218^ reported the preparation of the multicomponent high-entropy alloy (HEA) of CoCrFeMnNi nanomaterials with optimized stoichiometry with a smaller diameter (less than 5 nm) through PLAL. The HEA materials consist of multiple elements with homogeneous composition, and the composites of HEA were assessed for water oxidation, which exhibited a mass activity of 0.104 Amg^−1^. Nishi et al.^219^ reported CoO-Co_2_O_3_-Co(OH)_2_ prepared by femtosecond laser ablation for OER and suggested that the 5-nm CoO showed high mass activity with lower overpotential than that of submicron-sized Co-CoO in neutral solution, which was attributed to CoO_*x*_ electronic structure modification by laser ablation. Yu et al.^220^ reported laser fragmentation-induced defect-rich CoO with a 5 nm size for OER activity. The particles have fragmented from 8 to 4 nm due to the oxidation process in water where oxidative species are present. The CoO exhibited high OER activity in alkaline media with a current density of 10 mA cm^−2^ at 369 mV and a Tafel slope of 46 mV dec^−1^ ascribed to the large surface area, defects, and improved charge transfer rate. Hunter et al.^221^ prepared a series of Ni-Fe-based layered double hydroxides with nitrate and water as intercalating material [Ni_1–*x*_Fe_*x*_(OH)_2_](NO_3_)_*y*_(OH)_*x*−*y*_·*n*H_2_O via PLAL for water oxidation. It was noticed that the addition of Ti^4+^ and La^3+^ improves electrocatalytic activity with a lower overpotential of 260 mV at 10 mA cm^−2^. Zhou et al.^222^ prepared oxygen vacancy-induced Co_3_O_4_ NPs through laser fragmentation, which exhibited better water absorption ability and conductivity than the conventional RuO_2_, resulting in improved OER activity. Meng et al.^223^ synthesized CoOOH nanosheets with more oxygen vacancy through the PLAL process and used them as effective OER catalysts. Thus, creating more oxygen vacancies in the MO can enhance the conductivity and thin CoOOH nanosheets, leading to the availability of more active sites resulting in considerably improved OER activity of the CoOOH nanosheets.

Kang et al.^224^ reported laser synthesis of SAC on MWCNT for OER activity. Atomic Co and P are incorporated on the surface of MWCNT, where Co and P optimize the adsorption energy and improve charge transfer efficiency, resulting in superior OER performance than that of the RuO_2_ catalyst. Laser-synthesized FeO_*x*_ nanoparticles supported on the ITO substrate for OER activity were reported by Pizzolato et al.^225^. Thus, the FeO_*x*_@ITO electrode showed an excellent OER activity in neutral media with 0.58 V overpotential and quantitative FE. Hunter et al.^1^ studied the interlayer anions effect on the [NiFe]-LDH nanosheet prepared by PLAL for water oxidation activity and suggested that, in alkaline media, carbonate replaces other interlayer anions, resulting in enhanced catalytic activity. The oxidation activity was related to the p*K*_a_ of the conjugate acid of the interlayer anions. CoO nanocluster/CoFe LDHs hybrid prepared by PLAL showed a better OER activity^226^. Engineering the interfacial junction of hybrid catalysts often contributes to the synergistically improved electrocatalytic performance. The strong chemical coupling between the hybrid at the interface structure activates charge transfer from Co(II) of the oxide to Fe(III) in the LDHs through interfacial Fe-O-Co bond, resulting in the formation of a high oxidation state, Co(III), in the hybrid. The CoO/CoFe LDHs showed a synergistic effect for OER activity with a lower overpotential in alkaline media than that of the Ru/C electrocatalyst. Vassalini et al.^227^ reported Au–Fe nanoalloys prepared through PLAL, and this method incorporated around 11% of Fe into the Au lattice, which is not possible in any other method. Au–Fe nanoalloys exhibited high OER activity with lower overpotential in alkaline aqueous solution, which was attributed to the nanoalloying.

### ORR

Oxygen reduction reaction involves the conversion of chemical energy associated with the fuel and oxidant, generally O_2_ into electricity. ORR involves multiple electron transfer and is a kinetically slow reaction, although oxygen is a strong oxidant. The electrocatalysts developed for ORR reaction by PLA are discussed in this section. Ribeiro et al.^228^ prepared an MOF-derived PtCo/Co_3_O_4_ hybrid catalyst prepared through PLAL for ORR. The nanocrystal synthesized from 125 mg L^−1^ K_2_PtCl_4_ solution exhibits a five-fold increment in their specific mass activities in comparison to state-of-the-art commercial Pt catalyst due to the synergetic effect between the graphitic shell-coated bimetallic Pt–Co-NPs along with the electrochemically active Co_3_O_4_-covered carbon matrix support. Jindal et al.^229^ reported amyloid-β/Pt-NPs hybrid for ORR activity. The amyloid-β/Pt-NPs exhibited higher ORR activity with higher stability than that of commercial Pt/C, which was ascribed to the well-dispersed Pt-NPs on amyloid-β, resulting in the Pt-N bond between the amine group on the side chain and Pt, which acts as active sites for ORR reaction. Kohsakowski et al.^230^ reported for the first-time surfactant-free Pt nanomaterial by PLAL, which exhibited a high particle size of 6.6 nm compared to commercial Pt/C of 3.8 nm, and applied it to a proton exchange membrane fuel cell. The Pt showed high stability caused by the decreased Pt dissolution rate. Hu et al.^231^ reported the synthesis of PtCo nanoalloys using laser ablation in solution-galvanic replacement reaction (LAS-GRR), which resulted in uniformly alloyed cores with shells consisting of few nanometer Pt. The PtCo nanoalloys exhibited significant electrocatalytic activity for ORR in acid electrolyte with a three- and six-fold enhancement in mass and specific activities, respectively, which was attributed to the tandem LAS-GRR method to alter size distribution and compositional ratios and alloying of PtCo without surfactants or reducing agents. Furthermore, the same group reported the synthesis of PtCuCo ternary alloy through LAS-GRR as a surfactant-free synthesis route with an elemental composition of 72:12:16 (Pt:Co:Cu). Thus, the fabricated ternary alloy showed the best ORR activity owing to the incorporation of Cu, shift back the Pt d-band in the optimal position between Pt and PtCo binary alloys, hence tuning the binding affinities for oxygen and oxygenated species. PtCuCo possessed a core-shell structure with a shell consisting of largely Pt and a small amount of Cu with a core of PtCuCo alloy^232^. Kim et al.^233^ prepared LaMnO_3+δ_ NPs (LMO) through PLAL for ORR application. LMO showed ORR activity similar to commercial Pt/C attributed to the crystalline perovskite structure and enhanced surface area. LMO showed 20 and 2.2 times higher mass and specific activities when compared to bulk LMO. Ghobadi et al.^234^ prepared VSe_2_ 2D nanosheets and 3D NPs using PLA followed by liquid-phase exfoliation. The study suggests that the ORR activity of both NSs and NPs involves ∼2.85 number of electrons with a Tafel slope of 120 mV dec^−1^ in alkaline and neutral pH. In alkaline media, NPs exhibited better activity for OER with an onset potential of 1.5 V, whereas nanosheets showed better activity for HER with an onset potential of −0.25 V, and VSe_2_ NPs exhibited better activity in acidic media compared to nanosheets. Thus, the study confirms variation in the electrochemical activity of VSe_2_ nanosheets and NPs at different pH conditions. Brandiele et al.^235^ reported Pd_3_Y alloy synthesized by laser ablation in pure organic solvents (ethanol or methanol or hexane) as an ORR electrocatalyst. The Pd_3_Y exhibited high activity in terms of mass activity, half-wave potential, and specific activity than Pd/C and Pt/C. Also, the same group reported Pt_*x*_Y^236^ nanoalloys with superior mass activity (483 A g^−1^) and specific activity (0.562 mA cm^−2^) toward ORR application.

Highly active ORR catalysts are prepared through pulsed laser methods, such as Pt-based nanomaterials, PtCo/Co_3_O_4_ hybrid, amyloid-β/Pt nanoparticles, PtCo nanoalloys, PtCuCo, LaMnO_3+δ_ NPs, and Pd_3_Y alloys. Those nanomaterials showed high activity than highly active Pd/C and Pt/C catalysts ascribed to homogeneous size distribution, optimum composite composition ratios, and high surface area.

In addition to the photo/electrocatalytic applications, the materials produced via laser-assisted techniques are also used in many other fields. For example, Sportelli et al. reported the developments of Ag-NPs using PLA process and used for the antimicrobial studies^237^, Bagga et al. synthesized carbon NPs with controlled aqueous stability and viscosity using PLA of graphite target in DI water, which was used in printing applications^238^. ElFaham et al. fabricated Cu_2_O NPs via PLA for optoelectronic applications^239^. Apart from this, the lasers are extensively used for the following: (1) material manufacturing processing (drilling, cutting, cladding, welding, surface modification, hardening, engraving, marking, micromachining, and lithography), (2) medical applications (eye vision correction and surgery, dentistry, cancer photodynamic therapy, and cosmetic usage such as hair and tattoo removal, (3) laser projection displays, and (4) data storage.

## Summary and future perspectives

The rapid development of nanoscience and nanotechnology has resulted in the extensive applications of nanomaterials in various energy, environmental, and biology. The majority of commonly used conventional synthetic methods require using expensive precursor materials and involve ligand exchange reactions. Other disadvantages of the usual approaches are the formation of impurities and aggregates as well as the need for stabilizing agents, toxic reagents, or surfactants. To get rid of the complications faced in conventional methods, recently, pulsed laser-assisted methods such as PLA and PLI in the liquid medium have been increasingly used for preparing different compositions of nano- and submicron-sized products, including alloys, metals, carbon-based composites, and semiconductors. The main advantages of pulsed laser methods over conventional chemical synthesis techniques include tailoring the properties of targeted materials (i.e., surface structure, size, composition, and crystallinity) by tuning experimental conditions (e.g., laser wavelength, power, reaction time, and solvent). Moreover, pulsed laser approaches do not impose the use of stabilizing agents or surfactants, generate no byproducts, and offer fast and environmentally safer techniques. In this review, we briefly presented the research overview, fundamental aspects, and importance of the pulsed laser process, i.e., various roles and reported mechanisms involved in the production of various categories of nanomaterials, such as MNPs, oxides, non-oxides, and carbon-based materials. We then covered the up-to-date advancements in photo- and electrocatalytic nanomaterials via pulsed laser-assisted methodologies with detailed mechanistic insights and the structural optimization/regulation along with effective catalytic performances in various energy (HER, OER, and ORR) and environmental remediation (CO_2_ reduction into value-added products and photocatalytic wastewater treatment) processes.

Furthermore, several practical and fundamental issues of PLA/PLI-based metal NPs for efficient photo/electrocatalysis should be comprehended. Several aspects of this framework are discussed below.

1. Pilot-scale production of nanomaterials remains a limiting factor for pulsed laser techniques. More advancement in the design of the experimental arrangement is required.
2. The pulsed laser techniques mostly involve the production of metal, metal-oxide, and alloy NPs, although several recent works focused on the production of nonoxide materials, such as metal sulfides. There is much space for the extension of these techniques for the production of metal carbides, nitrides, and selenides because these materials are mostly prepared via solid-gas phase reaction or high-temperature programmed reaction.
3. As we know that the PLA process in liquid medium is free from surfactants or reducing agents, and recently few reports available for the production of metal sulfides and carbon composites without any additional source for sulfur and carbon. Thus, the solvents used in the PLAL itself act as a sulfur (e.g., DMSO) and carbon source (e.g., carbon-rich solvents like hexane). This should be extended to a wide range of metal sulfides and other nonoxide materials such as carbides and nitrides.
4. Conducting coordination polymers, inorganic complexes, and MOF-structured materials can be produced in a short time. Hence, mechanistic aspects should be explored in more detail.
5. Automation of pulsed laser system in the ablation of the desired target without aging the target can provide uniform and more amount of nanomaterials.
6. Integration of pulsed laser techniques with other synthetic routes, i.e., PLI with ultrasonochemical process can improve the rate of reaction, production, and uniformly distributed particles by the synergistic effect. Likewise, dual- or multilaser systems with combinations of PLA-PLI or PLA-PLA can be designed for the production of defect-rich and highly functional materials.
7. Real-time analysis of the produced NPs achieved by using in-situ XRD, FT-IR, Raman, PL, and UV-vis techniques should be performed for understanding detailed formation mechanisms.
8. As the solvents decompose during the PLA or PLI process and produce various free radicals and fragments, which can involve in the production of materials, some gaseous products can also be produced and utilized during solvent decomposition.

The above factors undoubtedly indicate that more research is required to advance pulsed laser techniques toward the realization of various applications. This review can provide practical guidance for future design and fabrication of innovative pulsed laser-induced nanomaterials with fascinating properties for advanced catalysis and beyond. Furthermore, it can contribute to igniting cross-disciplinary research among various scientific communities in the areas of physics, chemistry, biology, materials science, and technology.

## Data Availability

The data that support this research’s findings are available and can be provided based on the request to the corresponding authors.
